# Unrevealing the leaf frogs Cerrado diversity: A new species of *Pithecopus* (Anura, Arboranae, Phyllomedusidae) from the Mato Grosso state, Brazil

**DOI:** 10.1371/journal.pone.0184631

**Published:** 2017-09-27

**Authors:** Isabelle Aquemi Haga, Felipe Silva de Andrade, Daniel Pacheco Bruschi, Shirlei Maria Recco-Pimentel, Ariovaldo Antonio Giaretta

**Affiliations:** 1 Laboratório de Taxonomia e Sistemática de Anuros Neotropicais (LTSAN), Faculdade de Ciências Integradas do Pontal (FACIP), Universidade Federal de Uberlândia (UFU), Ituiutaba, Minas Gerais, Brazil; 2 Programa de Pós-Graduação em Biologia Animal, Instituto de Biologia, Universidade Estadual de Campinas (UNICAMP), Campinas, São Paulo, Brazil; 3 Laboratório de História Natural de Anfíbios Brasileiros (LaHNAB), Departamento de Biologia Animal, Instituto de Biologia, Universidade Estadual de Campinas (UNICAMP), Campinas, São Paulo, Brazil; 4 Departamento de Biologia Estrutural e Funcional, Instituto de Biologia, Universidade Estadual de Campinas (UNICAMP), Campinas, São Paulo, Brazil; 5 Departamento de Genética, Setor de Ciências Biológicas, Universidade Federal do Paraná (UFPR), Curitiba, Paraná, Brazil; Universita degli Studi di Roma La Sapienza, ITALY

## Abstract

The Neotropical frog genus *Pithecopus* comprises currently 10 species. A recent molecular phylogeny suggested the existence of two subclades within it, one of them including *P*. *palliatus*, *P*. *azureus*, *P*. *hypochondrialis*, and *P*. *nordestinus* (lowland species). Herein we describe a new species of this subclade from Pontal do Araguaia, in the Brazilian Cerrado in the Mato Grosso state. Recognition of the new species is supported by adult morphology, advertisement call and molecular data. The new species differs from *Pithecopus* highland species by its smaller head width and lack of the reticulate pattern on flanks. From lowland species, the new form differs by being significantly smaller in snout vent-length, advertisement call with the greatest number of pulses, and high genetic distance. Interestingly, we also report on occurrence of *P*. *hypochondrialis* (its sister species) at an adjacent site (about 3km). Also, we report on the occurrence of the new species in the Chapada dos Guimarães and Santa Terezinha, both also in the Mato Grosso state.

## Introduction

The increase of the fieldwork sampling efforts and the use of multiple databases in taxonomical studies open the doors to the fascinating biological diversity present in the central region of Brazil [1]. The Cerrado domain occupies the largest portion of central Brazil, and is composed by seasonal savannas with corridors of mesic gallery forests [2]. The increasing in anuran species richness recognized to Cerrado have been grown fast in the last decades and pointed to that species composition of this area need to better documentation [1]. In contrast, the Cerrado domain is categorized among the most threatened hotspots on the Earth, mainly due habitats loss by urban and agricultural development [3]. Therefore, to document this incredible diversity is a priority action to conservation of this Biome.

The *Pithecopus* genus Cope, 1866 was recently removed from the synonymy of *Phyllomedusa* Wagler, 1830 based on molecular data by Duellman et al. [4] that moved the species of the former *Phyllomedusa hypochondrialis* species group [5] to this revalidated genus. As presently defined, this genus comprises ten tropical South America species occurring east of the Andes from southern Venezuela to northern Argentina [4,6]. Phylogenetic inferences by Faivovich et al. [5] and Bruschi et al. [7] point to two well-supported subclades within the *Pithecopus*, one of which including essentially lowland species: *Pithecopus palliatus* (Peters, 1873), *P*. *azureus* (Cope, 1862), *P*. *hypochondrialis* (Daudin, 1800), and *P*. *nordestinus* (Caramaschi, 2006) [8]. This species group can also be characterized by the absence of a reticulate pattern on flanks, by a truncate snout tip, finger pads poorly developed, thumb equal or shorter than toe II and the finger I longer than the toe II [8,9].

Historically, *P*. *azureus* and more recently *P*. *hypochondrialis* [7] have been associated with Cerrado domain. However, Bruschi et al. [7] reported the difficulties to delimited these two species only based on those diagnose characters propose by Caramaschi [8], due overlaps of their phenotypic traits. According to Bruschi et al. [7], *P*. *azureus* is restrict to Paraguay and adjacent localities in Pantanal domain; they also extended the geographic distribution of *P*. *hypochondrialis* to the Cerrado domain, previously known only in the Amazon Rainforest. In addition, given the similarities among the advertisement calls of *P*. *azureus*, *P*. *hypochondrialis*, and *P*. *nordestinus*, Haga et al. [10] suggested that acoustic traits, at least solely, should not be employed as reliable diagnostic characters among these species.

Recently, we collected specimens from one population of *Pithecopus* in the middle portion of the Araguaia River in Brazilian Cerrado (Pontal do Araguaia) that aroused questions about their taxonomical status. To solve this, we combined both phenotypic (morphological and bioacoustical) and genotypic evidence, and concluded that no available name could be applied to this population, so we describe it herein as a new species of *Pithecopus*, belonging to the lowland species group.

## Material and methods

### Ethics statement

The individuals examined were collected under authorization number #30059–7 issued by SISBIO/ Instituto Chico Mendes de Conservação da Biodiversidade. The individuals collected were euthanized using anesthetic application (5% Lidocaine) to the skin to minimize animal suffering, according to recommendations of the Herpetological Animal Care and Use Committee (HACC) of the American Society of Ichthyologists and Herpetologists (available at: http://www.asih.org/publications), and approved by SISBIO/Institute Chico Mendes de Conservação da Biodiversidade as a condition for the concession license. Types are deposited in the Collection of frogs of the Museu de Biodiversidade do Cerrado (AAG-UFU), Universidade Federal de Uberlândia (UFU), Uberlândia (Brazil). Our new species hypothesis was written under the General Lineage Concept [11], which treats species as separately evolving metapopulation lineages.

### Taxa and specimens examined

**Fig 1:**
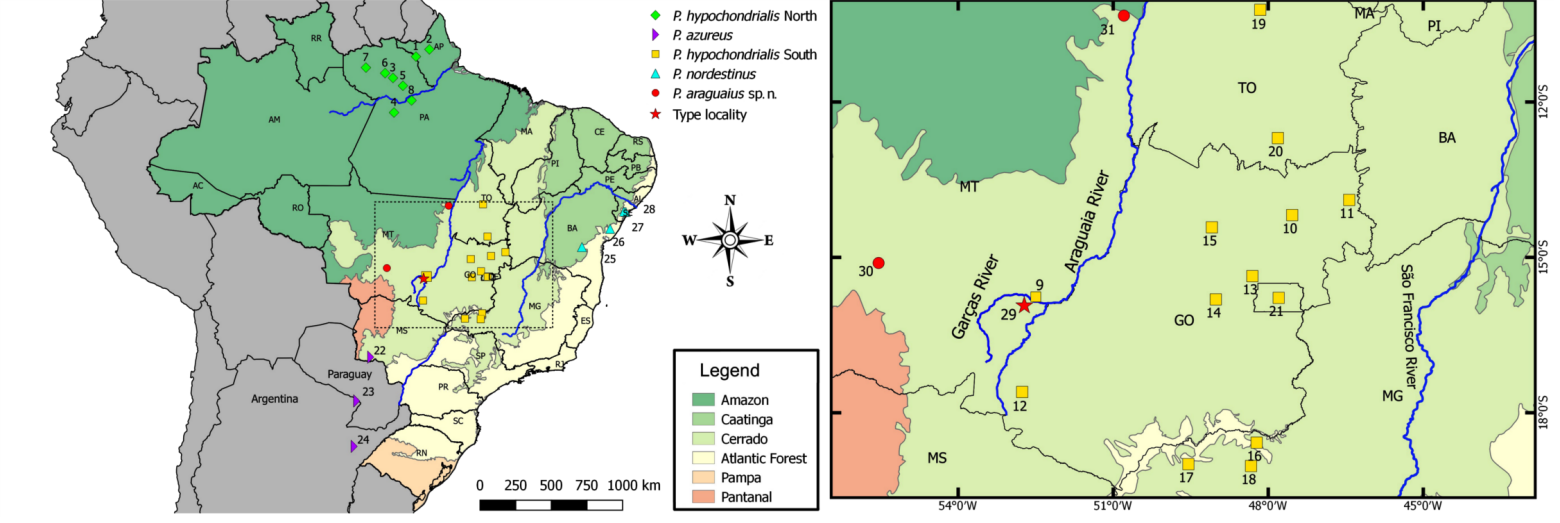
Map of South America showing the Brazilian domains and samples included in our molecular, morphological and acoustic comparisons. Note the type-locality of *Pithecopus araguaius* sp. n. (red star), Pontal do Araguaia, Mato Grosso state, and other localities for which the new species is know from. The inset shows the sample collected in the Barra do Garças (about 3 km from the type locality of the new species). Municipalities: (1) Laranjal do Jari—AP; (2) Serra do Navio—AP; (3) Alenquer—PA; (4) Belterra—PA; (5) Monte Alegre—PA; (6) Óbidos—PA; (7) Oriximiná - PA; (8) Prainha—PA; (9) Barra do Garças—MT; (10) Alto Paraíso de Goiás (Chapada dos Veadeiros)—GO; (11) Guarani de Goiás—GO; (12) Mineiros—GO; (13) Padre Bernardo—GO; (14) Pirenópolis—GO; (15) Uruaçu—GO; (16) Araguari—MG; (17) Ituiutaba—MG; (18) Uberlândia—MG; (19) Palmas—TO; (20) Paranã - TO; (21) Brasília—DF; (22) Bela Vista—MS; (23) Assunción—Paraguay; (24) Corrientes—Argentina; (25) Maracás—BA; (26) Alagoinhas—BA; (27) Areia Branca—SE; (28) Laranjeiras—SE; (29) Pontal do Araguai—MT; (30) Chapada dos Guimarães—MT; (31) Santa Terezinha—MT. Brazilian states: Acre—AC; Alagoas—AL; Amapá - AP; Amazonas—AM; Bahia—BA; Ceará - CE; Distrito Federal—DF; Espírito Santo—ES; Goiás—GO; Maranhão—MA; Mato Grosso—MT; Mato Grosso do Sul—MS; Minas Gerais—MG; Pará - PA; Paraíba—PB; Paraná - PR; Pernambuco—PE; Piauí - PI; Rio de Janeiro—RJ; Rio Grande do Norte—RN; Rio Grande do Sul—RS; Rondônia—RO; Roraima—RR; Santa Catarina—SC; São Paulo—SP; Sergipe—SE; Tocantins—TO.

Eighteen individuals of the new species used as types were collected in the municipality of Pontal do Araguaia (around 15°57’ S, 52°20’ W; 433 m above sea level; datum = WGS84), Mato Grosso (MT) state, Brazil (Fig 1).

Types and/or vouchers are deposited in the Museu de Zoologia (ZUEC), at Universidade Estadual de Campinas (UNICAMP); Célio F. B. Haddad amphibian collection (CFBH) at the Universidade Estadual Paulista (UNESP), Rio Claro, both in São Paulo state; Museu Nacional do Rio de Janeiro (MNRJ), at Universidade Federal do Rio de Janeiro (UFRJ), Rio de Janeiro, Rio de Janeiro state; and in collection of frogs of the Museu de Biodiversidade do Cerrado (AAG-UFU), Universidade Federal de Uberlândia (UFU), Uberlândia, Minas Gerais state, all in Brazil. Sixteen adult males from Chapada dos Guimarães and three adult males from Santa Terezinha (Fig 1) of the new species were also measured as additional specimens. The complete list of vouchers examined is available on S1 File (Appendix A).

Because the new species most resembles *P*. *hypochondrialis* and this taxa have a large geographical distribution in Brazil with morphological variation already reported [5,7], we considered a large number of specimens from several localities in our analyses. We treated separately specimens of *P*. *hypochondrialis* from Pará and Amapá as “*P*. *hypochondrialis* North” because their proximity to Suriname (type-locality of *P*. *hypochondrialis)* and the specimens from Minas Gerais, Goiás, Mato Grosso, and Tocantins as “*P*. *hypochondrialis* South”, according to genetic differences pointed out by Bruschi et al. [7] and intrageneric relations recovered in our mitochondrial topology (see results section). Our sample of “*P*. *hypochondrialis* South” contains specimens, calls and mtDNA sequences from Barra do Garças, Mato Grosso state (Fig 1), a neighboring locality to the type-locality of the new form. Details for examined specimens in S1 File (Appendix A).

### Morphometry

Morphometric traits of adult males were measured using a Mitutoyo Absolute digital caliper CD-6” CSX to the nearest 0.1 mm, except for finger and toe discs which were measured under a stereomicroscope (Zeiss Stemi 2000) coupled to an ocular micrometer.

Twelve morphometric traits were measured following Watters et al. [12]: snout-vent length (SVL), hand length (HAL), forearm length (FAL), thigh length (THL), foot length (FL), head length (HL), head width (HW), eye diameter (ED), internarial distance (IND), tibia length (TL) (= shank length), tympanum diameter (TD), and eye-nostril distance (END). Two morphometric traits follow Heyer et al. [13]: upper arm length (UAL) and tarsus length (TAL). The disc diameters of third finger (3FD), fourth finger (4FD), fourth toe (4TD), and fifth toe (5TD) were measured according to Watters et al. [12] (see their Fig 3P). The axilla-groin length (AGL) was measured according to Clemente-Carvalho et al. [14].

For comparisons were measured 21 types (holotype + paratopotypes) plus 31 ordinary specimens of *Pithecopus nordestinus* from Alagoinhas (BA), Areia Branca and Laranjeiras (both from Sergipe state); and 13 topotypes of *P*. *azureus* from Asunción, Pararaguay (type locality [8]), one ordinary specimen from Corrientes, Argentina, and 11 from Bela Vista (MS) (our nearest acoustic sample from its type-locality). We also examined specimens of *P*. *palliatus*, *P*. *rohdei* (Mertens, 1926), *P*. *ayeaye* Lutz, 1966, *P*. *megacephalus* (Miranda-Ribeiro, 1926), *P*. *centralis* (Bokermann, 1965), *P*. *oreades* (Brandão, 2002), and *P*. *rusticus* (Bruschi, Lucas, Garcia, and Recco-Pimentel, 2015). *Pithecous araguari* (Giaretta, Oliveira, and Kokubum, 2007) is a taxonomically disputed species (A. A. Giaretta, in prep.) and, as the new form can be promptly distinguished from it by the same set of features regarding to the other highland *Pithecopus* species (e.g. *P*. *ayeaye*), detailed comparisons with the new form will not be reported here. Further details on examined specimens in S1 File (Appendix A) (see Fig 1).

### Bioacoustics

Vocalizations of the seven topotypic males were recorded between 20:40 to 03:00 h on three field trips (12–16 February 2010, 6–10 January 2014, and 1–5 December 2014; air temperature 24–26°C and water 26–31°C), totalizing fifty-four advertisement calls. Call vouchers: AAG-UFU 3444, 3449, 4877–78, and 5042. The recordings (wav format) were obtained with the following digital recorders: Marantz PMD 671 (Marantz, Japan), a Boss 864 8-Track Digital Studio (Boss Corporation, Japan), both coupled to Sennheiser ME67/K6 microphones (Sennheiser electronic GmbH & Co., Germany); and M-audio Microtrack II (M-audio, USA) coupled to Sennheiser ME66/K6. Recorders were set at a sampling rate of 44.1–48.0 kHz and a resolution of 16 bits.

Acoustic comparisons were made among the lowland *Pithecopus* species. The complete comparative dataset includes advertisement calls of the *P*. *azureus*, described by Haga et al. [10], besides forty-four calls of two males *P*. *nordestinus* (from Areia Branca, Itabaiana and Muruim, SE), and 212 calls of 41 males of *P*. *hypochondrialis*, including original call data of *“P*. *hypochondrialis* North” (Serra do Navio, AP) and “*P*. *hypochondrialis* South” (from Barra do Garças, MT; Brasília, DF; Araguari and Uberlândia, MG; Padre Bernardo, Pirenópolis and Uruaçu, GO). Call vouchers of *P*. *hypochondrialis* are from Uruaçu: AAG-UFU 0991–93; Pirenópolis: AAG-UFU 0331–33; Barra do Garças: AAG-UFU 1082–83, 3489; Araguari: AAG-UFU 4832; and Serra do Navio: AAG-UFU 5998). Sound files are in the Arquivo Sonoro da Coleção de Anuros da Universidade Federal de Uberlândia at UFU and Fonoteca Neotropical Jacques Vielliard (FNJV) at UNICAMP. Call descriptions and the acoustic terminology follow Köhler et al. [15] and details for acoustic terminology employed here for the species of *Pithecopus* are available in S1 Table (Appendix B).

The calls of some species of *Pithecopus* often have a few weak isolated pulses at the end (more common) or at the beginning (unusual) (see results section). To compare call traits equivalently among specimens/species, we used the main (stronger) group of pulses of each call (hereafter called “core portion”) of the new species, *P*. *hypochondrialis*, and *P*. *nordestinus*. Traits such as pulse duration and pulses per second were measured within the core portion (further details in S1 Table—Appendix B). The presence or absence of isolated pulses were recorded for each individual; when they were absent, we state it as “0”, when at least one was present, as “1”, and two pulses as “2”. We calculated means and standard deviations (SD) for each individual and then the overall mean and SD was calculated based on those values, whereas the range encompassed the minimum and maximum values for the whole sample. We analyzed calls using Raven Pro 1.5, 64 bit version [16] with the following settings: window type = Hann, window size = 256 samples, 3 dB filter bandwidth = 270 Hz, brightness = 50%, contrast = 50%, overlap = 85% (locked), color map = “Cool”, DFT size = 1024 samples (locked), grid spacing (spectral resolution) = 46.9 Hz. We analyzed temporal traits in oscillograms and spectral traits in spectrograms [15]; we used the ‘Peak Frequency’ function to determine the peak of dominant frequency. We generate sound figures through package Seewave v.1.6 package [17], R platform (version 3.3.1) [18]. Seewave settings for the spectrograms were: Hanning window, 85% overlap, and 256 points resolution (FFT). Analyzed sound files are listed in S2 Table (Appendix C).

### Statistical analysis

Considering the morphological and acoustic (multivariate) datasets separately, we sought for discrimination between populations/species by applying two functions: (1) randomForest (RF) (radomForest v. 4.6–12 package) [19] and (2) DAPC (Discriminant Analysis on Principal Components, adegenet v. 2.0.1 package [20,21]. RandomForest algorithm constructs many (e.g. 500) classification trees using bootstrap samples of the data (each split using the best predictors among those randomly chosen at each node) then generating classifiers and aggregating results by voting to classes [19]. The classic Discriminant Analysis (DA) depends on multivariate normality [22] and on a larger number of objects than variables. The multivariate normality of the original data was evaluated through the function mardiaTest (MVN package) [23]. The DAPC performs analyses on the Principal Component scores [20,21]. The application of a DA on a few axes (preserving about 95% of the variance) of a Principal Component Analysis, as performed by DAPC, improves the imbalance between objects and traits [21]. Despite the lack of normality in both of our datasets (details not shown), the results of DAPC are presented within an exploratory context to assess the congruence between it and randomForest discriminations. The direct or indirect packages related the application of both discriminant functions were run in R.

For the multivariate analysis and statistical tests we used all the morphometric features detailed above. In addition to the types, additional specimens of the new form from Chapada dos Guimarães and Santa Terezinha (MT) were also considered. For the acoustic analyses we used: call duration (entire call), number of pulses, pulse duration, interpulse interval within core, core duration (main stronger group of pulses), pulses per core, pulses per second, isolated pulse and peak of dominant frequency. Considering that both multivariate analyses, to both datasets, were concordant in species discrimination (see Results section) we present the RF classification results in tables and DAPC in scatter plots.

The acoustic and morphometric traits were tested for statistical significance of the differences among population/species through the Exact Wilcoxon Mann Whitney Rank Sum Test, function wilcox_test of the package Coin (Resampling Statistics model) [24] in R. As these tests were done between species/populations pairs, the significance levels (“*p*”) were adjusted considering the number of pairings through the method of Holm (p.adust function in R).

### Molecular divergences

To evaluate the reciprocal monophyly of new species respect to the sister species/taxon, we performed phylogenetic analyses of mitochondrial 16S ribosomal sequences (1490 characters) of paratopotypes (ZUEC 21657, 21659 and 21660), and additional specimens (CFBHt 4638, 4684, 4687) of the new *Pithecopus* species. The data matrix was completed with sequences of at least one individual of each of the *Phyllomedusidae* species available in GenBank database. We also included in our dataset sequences of the individuals from *Pithecopus* from Pará and Amapá states (“*P*. *hypochondrialis* North”) and the specimens from Minas Gerais, Maranhão, Goiás and Tocantins states (“*P*. *hypochondrialis* South”), according the topology of Bruschi et al. [7]. We chose *Agalychnis granulosa* as the outgroup based on Faivovich et al. [5]. A list of species, sequences and GenBank accession numbers is provided in S3 Table.

Genomic DNA was extracted from liver or muscle tissue using the TNES method, as applied by Bruschi et al. [25]. The tissues are stored at -70°C in the tissue bank of the Departamento de Biologia Estrutural e Funcional at UNICAMP, São Paulo state, Brazil. The mitochondrial 16S ribosomal genes were amplified using the primers MVZ 59(L), MVZ 50(H), 12L13, Titus I (H), Hedges16L2a, Hedges16H10, 16Sar-L, and 16Sbr-H (for primer sequences, see [26]. The amplified PCR products were purified using Exonuclease I (10 units) and SAP (1 unit), with a 45-min incubation at 37°C and a 10-min denaturation at 85°C, then used directly as templates for sequencing in an automatic ABI/Prism DNA sequencer (Applied Biosystems, Foster City, CA, USA) with the BigDye Terminator kit (Applied Biosystems, Foster City, CA, USA), as recommended by the manufacturer. The DNA samples were sequenced bidirectionally and edited in the software CodonCode Aligner 3.7.1 (Codon Code Corporation, Dedham, MA, USA).

We aligned sequences using CLUSTALW [27] implemented in CodonCode Aligner 4.0. We used three different gap penalties (5, 10, and 15) for each gene to identify potential sites of ambiguous homology [28]. Gap length was kept constant (0.20) and all other parameters were set at default settings. The dataset was used to phylogenetic reconstruction by Bayesian inferences (BI) and Maximum Parsimony criterion.

Bayesian inference was based on a Markov chain Monte Carlo (MCMC) analysis conducted in MrBayes 3.1.2 [29] with two independent runs, each with four chains and sampling every 1000 generations for six million generations. An adequate burn-in (the first 25% trees were excluded) was determined by examining a plot of the likelihood scores of the heated chain for convergence and stationarity. The evolutionary model most appropriate (GTR+R+I) was selected by MrMODELTEST [30] using the Akaike Information Criterion (AIC). The trees were sampled every 100 generations, excluding the first 25% of the trees as burn-in, determined by examining a plot of the likelihood scores the heated chain for convergence and stationarity. Tracer software version 1.5 [31] was used to confirm the quality of the parameters of the Bayesian inferences. To the Maximum Parsimony criterion was implemented in TNT v1.1 software [32] using a heuristic search method with tree bisection-reconnection (TBR) swapping and 100 random additional replicates. The bootstrap values of the branches inferred in this analysis were calculated with 1000 non-parametric pseudoreplicates.

Uncorrected genetic distances (*p*-distances) among the sequences of the *Pithecopus* species were calculated using the maximum composite likelihood model [33] implemented in MEGA5 [34]. Gaps and missing data were eliminated in this analysis and all parameters were left as in the default settings.

To evaluate if geographic distances could explain the genetic differences among populations from Pontal do Araguaia + Chapada dos Guimarães and Barra do Garças, we performed a Mantel test in Alleles In Spaces (AIS) software. For identifying biogeographic boundaries or areas with largest genetic differences among samples collected in the Mato Grosso state and allocated in two different clades in our phylogenetic inferences, we compute the Monmonier’s algorithm of maximum-differences [35] in Alleles In Spaces (AIS) software.

### Nomenclatural acts

The electronic edition of this article conforms to the requirements of the amended International Code of Zoological Nomenclature, and hence the new names contained herein are available under that Code from the electronic edition of this article. This published work and the nomenclatural acts it contains have been registered in ZooBank, the online registration system for the ICZN. The ZooBank LSIDs (Life Science Identifiers) can be resolved and the associated information viewed through any standard web browser by appending the LSID to the prefix “http://zoobank.org/”. The LSID for this publication is: urn:lsid:zoobank.org:pub:00E56B5F-1EBD-47C8-8A54-2EC50EAD70A9. The electronic edition of this work was published in a journal with an ISSN, and has been archived and is available from the following digital repositories: PubMed Central, LOCKSS.

## Results

### Description of the new species

**Fig 2:**
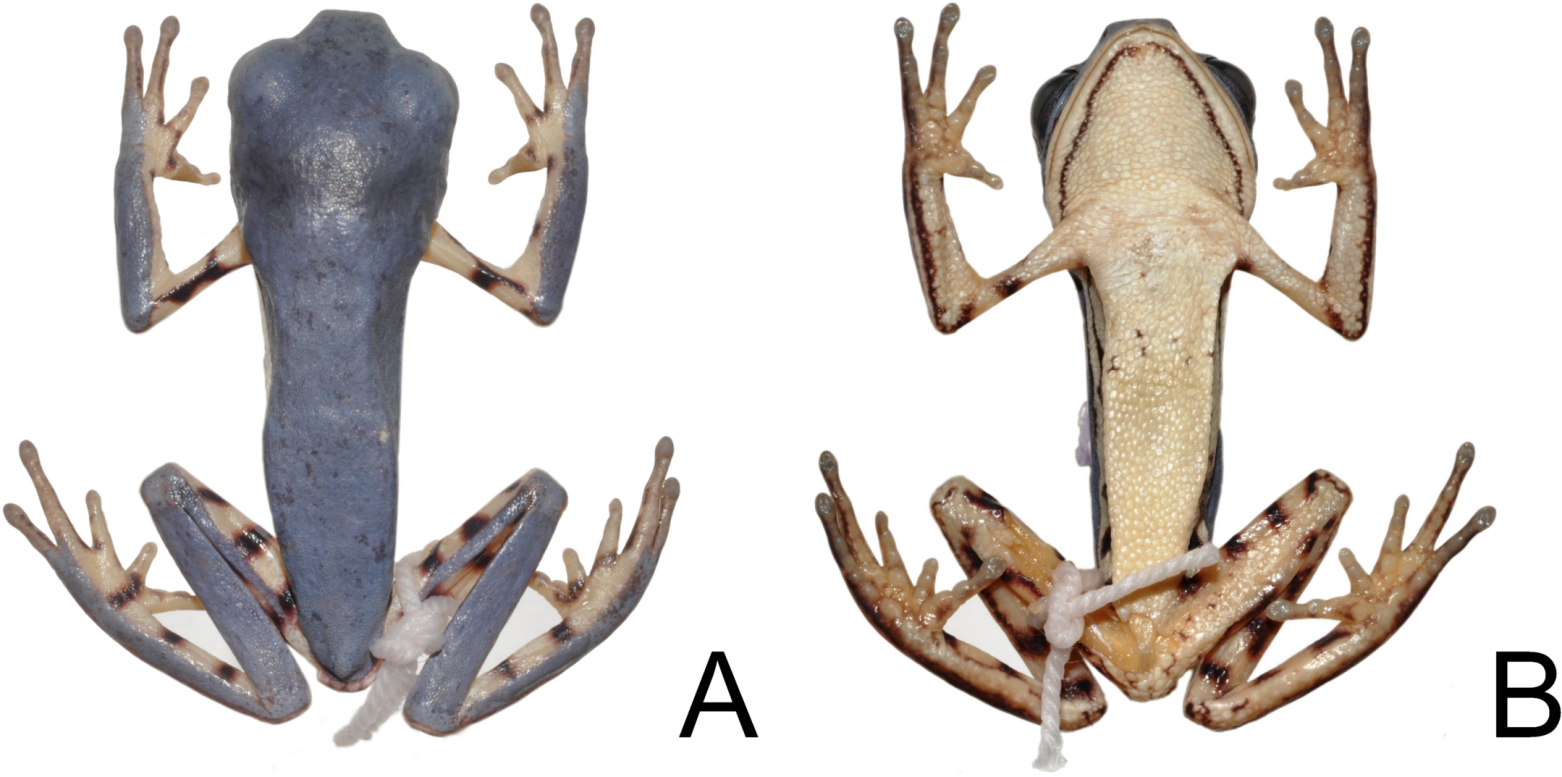
*Pithecopus araguaius* sp. n. holotype (AAG-UFU 3444), an adult male. Dorsal (A) and ventral (B) views. SVL = 30.6 mm.

**Fig 3:**
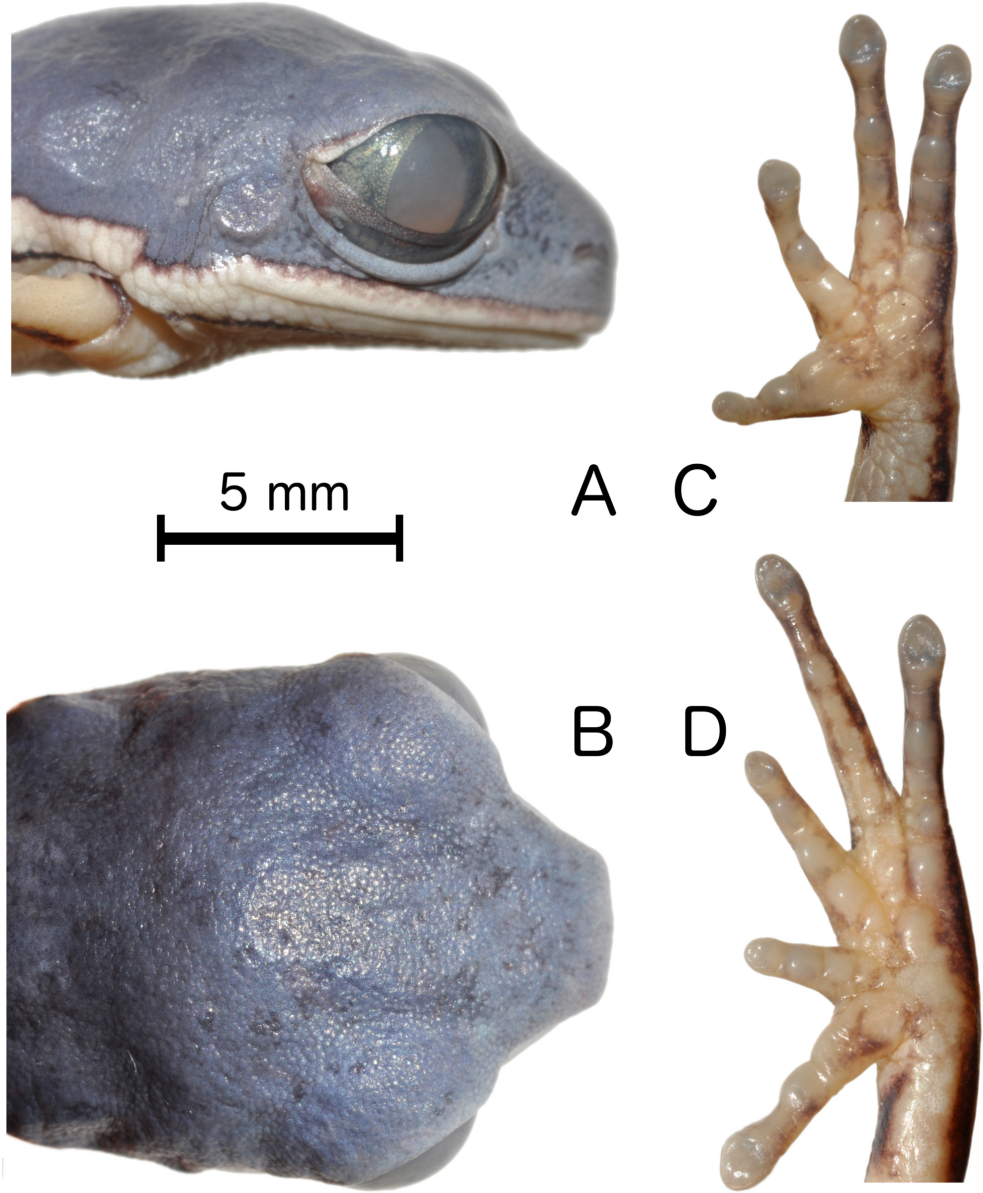
*Pithecopus araguaius* sp. n., adult male, holotype (AAG-UFU 3444). (A) Lateral and (B) Dorsal views of head; ventral views of (C) hand and (D) foot. Scale bar = 5mm.

***Pithecopus araguaius* sp. n.** urn:lsid:zoobank.org:act:9F062EDB-9B50-4EB7-A797-6CE5AD79CB53.

(Figs 2 and 3; Tables 1 and 2)

**Table 1:** Morphometry of adult males of *Pithecopus araguaius* sp. n. (type series, Chapada dos Guimarães and Santa Terezinha), two populations of *P*. *hypochondrialis*, *P*. *nordestinus* (including holotype and 20 paratopotypes) and *P*. *azureus* (including 13 topotypes).

	*P*. *araguaius* sp. n.			*P*. *hypochondrialis*		*P*. *nordestinus*	*P*. *azureus*
Traits	Type-series	Chapada dos Guimarães	Santa Terezinha	North	South		
	n = 18	n = 15	n = 3	n = 43	n = 50	n = 52	n = 25
Snout-vent length	31.7 ± 1.2 (30.3–33.8)	31.8 ± 1.7 (28.3–33.7)	32.6 ± 1.2 (31.5–33.9)	37.2 ± 1.9 (33.6–42.0)	35.4 ± 1.5 (32.2–39.5)	36.3 ± 1.9 (32.9–42.5)	35.9 ± 1.9 (33.1–39.7)
Head length	6.8 ± 0.5 (6.1–8.0)	7.2 ± 0.4 (6.6–8.0)	7.0 ± 0.6 (6.4–7.4)	8.3 ± 0.7 (7.1–9.7)	7.8 ± 0.9 (5.9–9.9)	7.5 ± 0.7 (6.3–9.7)	7.6 ± 0.9 (6.0–9.8)
Head width	9.6 ± 0.3 (9.2–10.1)	9.5 ± 0.4 (8.6–10.2)	10.1 ± 0.2 (9.8–10.3)	11.6 ± 0.6 (10.5–13.0)	11.0 ± 0.6 (9.8–12.3)	11.1 ± 0.6 (9.9–12.7)	10.6 ± 0.5 (9.8–11.4)
Axilla-groin length	14.4 ± 1.3 (11.4–16.9)	15.3 ± 1.2 (13.5–17.5)	15.7 ± 0.7 (14.9–16.2)	17.9 ± 1.7 (14.7–22.2)	16.1 ± 1.5 (13.5–20.1)	17.3 ± 1.5 (14.5–20.8)	16.9 ± 1.6 (14.1–19.8)
Eye diameter	4.0 ± 0.2 (3.7–4.4)	4.0 ± 0.3 (3.5–4.4)	3.7 ± 0.2 (3.6–4.0)	4.5 ± 0.3 (3.6–5.3)	4.4 ± 0.3 (3.8–5.1)	4.2 ± 0.4 (3.3–4.9)	4.0 ± 0.3 (3.5–4.6)
Tympanum diameter	1.6 ± 0.2 (1.3–1.8)	1.6 ± 0.2 (1.2–2.0)	1.7 ± 0.1 (1.5–1.8)	1.8 ± 0.3 (1.4–2.3)	1.7 ± 0.3 (1.1–2.3)	1.8 ± 0.3 (1.2–2.3)	1.7 ± 0.2 (1.2–2.3)
Eye-nostril distance	2.0 ± 0.2 (1.7–2.3)	2.0 ± 0.1 (1.8–2.2)	2.1 ± 0.2 (1.9–2.2)	2.6 ± 0.2 (2.1–3.0)	2.4 ± 0.2 (1.9–2.9)	2.5 ± 0.2 (2.0–3.1)	2.3 ± 0.3 (1.9–2.9)
Internarial distance	2.8 ± 0.2 (2.5–3.2)	3.0 ± 0.3 (2.1–3.6)	3.5 ± 0.1 (3.4–3.6)	3.5 ± 0.2 (3.1–3.9)	3.3 ± 0.3 (2.8–3.9)	3.4 ± 0.3 (2.9–4.0)	3.5 ± 0.5 (2.7–4.3)
Upper arm length	6.9 ± 0.4 (6.1–7.8)	7.2 ± 0.8 (6.1–8.8)	6.8 ± 0.7 (6.4–7.6)	7.6 ± 0.4 (6.6–8.4)	7.3 ± 0.4 (6.6–8.3)	7.3 ± 0.5 (6.5–8.5)	7.1 ± 0.5 (6.1–8.5)
Forearm length	7.4 ± 0.4 (6.5–8.0)	7.6 ± 0.3 (6.8–8.0)	7.5 ± 0.4 (7.1–7.8)	8.7 ± 0.6 (7.5–9.8)	8.1 ± 0.5 (6.7–9.1)	8.2 ± 0.6 (6.9–9.3)	7.9 ± 0.8 (6.5–9.8)
Hand length	7.8 ± 0.4 (7.2–8.6)	8.0 ± 0.6 (6.8–9.0)	8.7 ± 0.2 (8.6–8.9)	9.7 ± 0.6 (8.3–10.7)	9.3 ± 0.5 (8.4–10.3)	9.4 ± 0.7 (8.0–11.2)	9.2 ± 0.7 (7.8–10.6)
Thigh length	13.4 ± 0.6 (12.3–14.4)	13.8 ± 0.9 (12.4–15.1)	14.4 ± 0.2 (14.2–14.6)	16.7 ± 0.9 (14.9–18.7)	14.9 ± 0.7 (13.2–16.3)	15.1 ± 0.8 (13.6–16.6)	14.7 ± 0.8 (12.8–16.2)
Tibia length	13.4 ± 0.5 (12.3–14.1)	13.6 ± 1.0 (11.5–15.9)	14.7 ± 0.4 (14.2–15.0)	16.6 ± 1.1 (14.8–19.3)	15.0 ± 0.7 (13.6–16.5)	14.8 ± 0.7 (13.4–16.2)	14.5 ± 0.8 (12.8–16.5)
Tarsus length	9.0 ± 0.6 (8.1–10.3)	9.2 ± 0.5 (8.5–9.9)	10.0 ± 0.2 (9.8–10.1)	11.5 ± 0.9 (9.9–13.7)	10.5 ± 0.5 (9.4–11.5)	9.8 ± 0.6 (8.8–11.1)	9.5 ± 0.6 (8.3–10.8)
Foot length	10.3 ± 0.4 (9.6–11.1)	10.6 ± 0.7 (9.3–12.0)	11.5 ± 0.1 (11.4–11.6)	12.5 ± 0.8 (10.7–14.1)	12.2 ± 0.5 (11.4–13.3)	11.7 ± 0.6 (10.4–13.1)	11.8 ± 0.7 (10.3–13.1)
Third finger disc diameter	0.8 ± 0.1 (0.7–1.0)	0.9 ± 0.1 (0.8–1.1)	1.1 ± 0.0 (1.1–1.1)	1.3 ± 0.2 (0.8–1.6)	1.0 ± 0.1 (0.7–1.3)	1.0 ± 0.2 (0.5–1.4)	1.0 ± 0.1 (0.7–1.2)
Fourth finger disc diameter	0.9 ± 0.1 (0.7–1.0)	1.0 ± 0.1 (0.8–1.1)	1.1 ± 0.0 (1.1–1.1)	1.2 ± 0.2 (0.8–1.6)	1.1 ± 0.1 (0.9–1.4)	1.0 ± 0.2 (0.6–1.3)	1.0 ± 0.1 (0.7–1.2)
Fourth toe disc diameter	0.9 ± 0.1 (0.7–1.2)	0.9 ± 0.1 (0.8–1.1)	1.0 ± 0.1 (1.0–1.1)	1.3 ± 0.2 (0.9–1.6)	1.1 ± 0.1 (0.8–1.4)	1.0 ± 0.2 (0.6–1.4)	1.0 ± 0.2 (0.7–1.5)
Fifth toe disc diameter	1.0 ± 0.1 (0.9–1.1)	1.0 ± 0.1 (0.8–1.2)	1.1 ± 0.1 (1.0–1.1)	1.4 ± 0.2 (0.9–1.7)	1.2 ± 0.1 (0.9–1.6)	1.0 ± 0.2 (0.7–1.5)	1.0 ± 0.2 (0.7–1.3)

Values in millimeters as mean ± standard deviation (minimum–maximum); n = number of measured specimens. See text for the statistical significance of the differences and multivariate comparative analysis.

**Table 2:** Advertisement call traits of three lowland *Pithecopus* species: *P*. *araguaius* sp. n. (topotypes); two populations of *P*. *hypochondrialis*; and *P*. *nordestinus*.

Call traits	*P*. *araguaius* sp. n. n = 7 (54 calls)	*P*. *hypochondrialis* North n = 8 (32 calls)	*P*. *hypochondrialis* South n = 33 (180 calls)	*P*. *nordestinus* n = 3 (54 calls)
Call duration (ms)	41.2 ± 5.0 (28–63)	26.5±2.4 (19–42)	39.1 ± 8.8 (22–72)	49.2 ± 12.7 (19–85)
Calls per minute	12.2 ± 9.3 (2.2–59.8)	19.5±8.3 (3.2–29.2)	10.1 ± 10.4 (1.3–95.8)	40 ± 10.2 (11–48)
Pulses/call	6.0 ± 0.6 (5–8)	3.8±0.4 (3–5)	4.2 ± 0.4 (3–6)	4.1 ± 0.3 (3–6)
Pulse duration (ms)	5.1 ± 1.3 (2–17)	5.8±1.0 (3–10)	7.0 ± 1.1 (2–15)	7.4 ± 0.5 (1–16)
Interpulse interval within core (ms)	1.8 ± 0.8 (0–4)	1.5±0.7 (0–4)	2.1 ± 0.9 (0–7)	0.8 ± 0.3 (0–4)
Core duration (ms)	39.3 ± 5.4 (28–48)	26.3±2.5 (19–31)	33.2 ± 3.2 (19–61)	24.5 ± 2.1 (17–33)
Pulses/core	6.0 ± 0.5 (5–8)	3.8±0.4 (3–4)	4.0 ± 0.2 (3–5)	3.1 ± 0.1 (3–4)
Duration of isolated pulses (ms)	3.5 (2–5)*	7.0 (7.0–7.0)*	8.0 ± 2.2 (3–15)	8.0 ± 1.7 (2–15)
Number of isolated pulses	1.0 (1–1)*	1.0 (1–1)*	1.0 ± 0.0 (1–1)	1.3 ± 0.2 (1–2)
Core and isolated pulse interval (ms)	6.0 (5–8)*	10 (10–10)*	12.5 ± 3.4 (5–21)	18.2 ± 7.0 (10–48)
Pulses per second	155 ± 20 (114–206)	145.7±14.5 (115–182)	121 ± 12.6 (66–160)	126.4 ± 10.9 (91–177)
Min. dominant frequency (Hz)	1445 ± 367 (1044–3029)	1359±126 (1038–1646)	1076 ± 219 (328–1592)	1493 ± 183 (1133–1897)
Max. dominant frequency (Hz)	3489 ± 409 (3088–4907)	2664±204 (2343–3403)	3398 ± 684 (2482–5374)	2563 ± 167 (2351–3124)
Peak of dominant frequency (Hz)	2540 ± 308 (2240–3316)	2110±101 (1809–2240)	2170 ± 145 (1781–2625)	2049 ± 6 (1969–2156)
Air temperature range	25 ± 0.8 (24–26)	26 ± 0.0 (26–26)	22.4 ± 2.1 (18–26)	25.8 ± 2.7(21–27)
Time	20:41h–2:53h	19:24 h–22:57 h	20:05 h–03:41 h	21:10 h–00:00 h

Mean+SD (minimum–maximum). n = number of recorded specimens (number of analyzed calls). See text for localities and multivariate comparative analysis.

* A single male had these traits.

*Phyllomedusa* cf. *hypochondrialis*: Bruschi et al. [7] (see their Table 2, L9)

#### Holotype

AAG-UFU 3444, adult male (Fig 2 and Fig 3), collected in the Municipality of Pontal do Araguaia (15°54’51.4”S, 52°16’1.53”W, 418 m above sea level), Mato Grosso state, Brazil, on 8 January 2014 by A. A. Giaretta, I. A. Haga, and F. S. Andrade.

#### Paratopotypes

**Fig 4:**
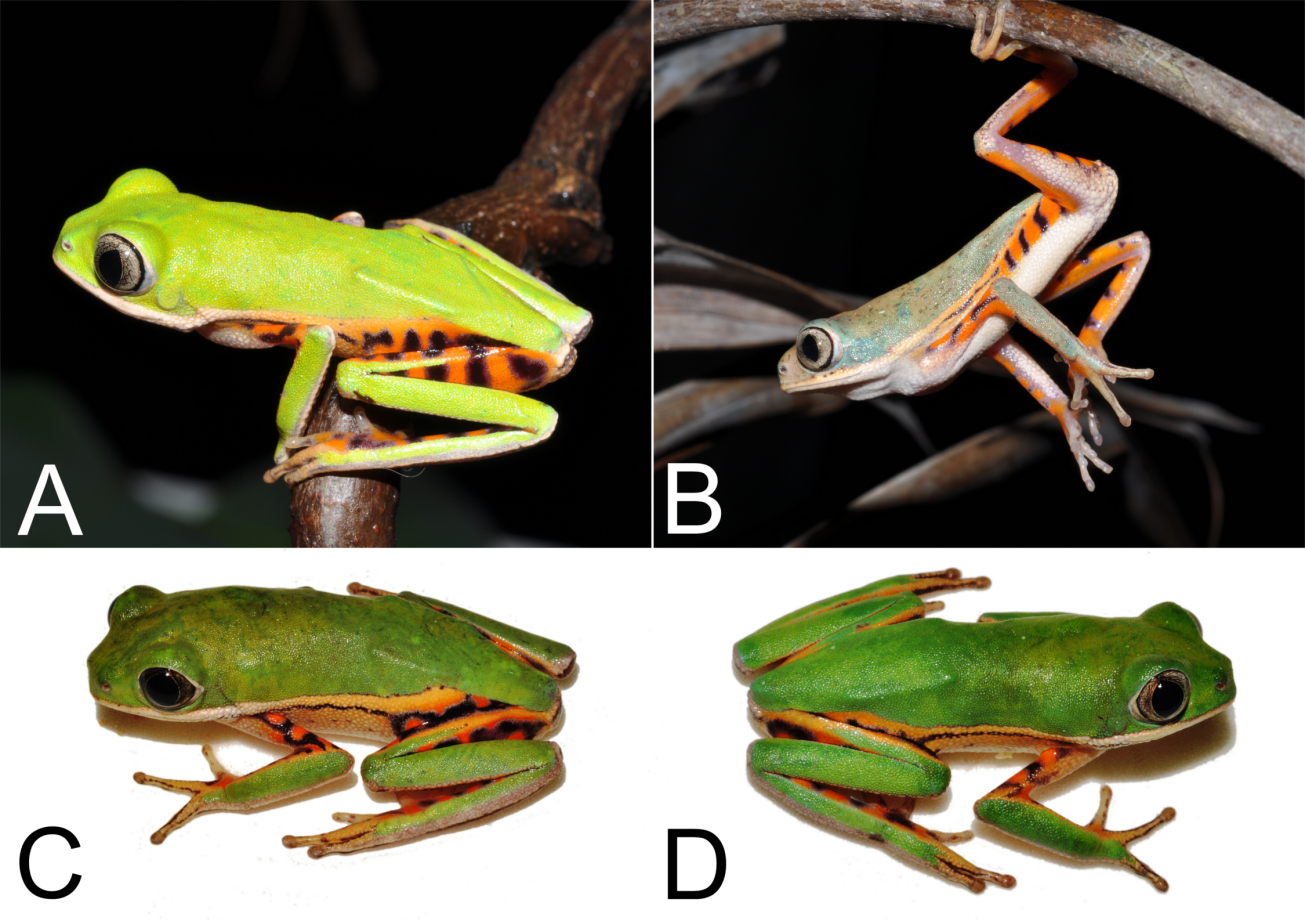
Paratopotypes of *Pithecopus araguaius* sp. n. in life. **Specimens from Pontal do Araguaia, Mato Grosso state, Brazil.** Adult males (A) AAG-UFU 4877, SVL = 31.4 mm; (B) AAG-UFU 4878, SVL = 33.6 mm; (C) AAG-UFU 3442, SVL = 32.8 mm and (D) AAG-UFU 3443, SVL = 31.0 mm.

Seventeen adult males (Fig 4): AAG-UFU 4877–4882 collected in February 2010 by A. A. Giaretta; AAG-UFU 3442–3443, 3445–3449, collected in January 2014 by A. A. Giaretta, I. A. Haga, and F. S. Andrade, and ZUEC 21657–60 collected in December 2014 by A. A. Giaretta, and C. S. Bernardes.

List of additional specimens: Brazil: Mato Grosso state: Chapada dos Guimarães: ZUEC 15884–15888, 21644–21645, 21647; CFBH 14321–14322, 14367–14370, 14406; Santa Terezinha: ZUEC 7457–7458, 13503.

#### Type locality

Pontal do Araguaia (15°54’51.4”S, 52°16’1.53”W, 418 m above sea level), Mato Grosso state, Brazil.

#### Diagnosis

*Pithecopus araguaius* sp. n. is assigned to the genus *Pithecopus* (former *Phyllomedusa hypochondrialis* species group) [8] by the following set of characters: (1) small body size; (2) dorsolateral macroglands (sensu [36]) indistinct; (3) smooth skin on back and granulose on belly; 4) fingers and toes long and slender with terminal discs poorly developed; (5) grasping (opposable to the others) finger I and toe I. The new species differs from the highland *Pithecopus* species by its (6) smaller head width; and (7) lack of the reticulate pattern on flanks; and from the lowland species by having significant differences in its (8) smaller snout vent-length, and (9) call with a greater number of pulses.

#### Comparison with others species

*Pithecopus araguaius* sp. n. is promptly distinguished from *P*. *ayeaye*, *P*. *centralis*, *P*. *oreades*, *P*. *megacephalus*, and *P*. *rusticus* by lacking the reticulate color pattern on flanks and by its smaller head width (8.6–10.5 vs. 11.0–14.5 mm, combined values). Also, *P*. *araguaius* sp. n. differs from *P*. *ayeaye*, *P*. *megacephalus*, *P*. *oreades*, and *P*. *rusticus* (8.5–14.0 mm, combined values) by its smaller head length (6.1–8.0 mm). The new species is distinguished from *P*. *centralis*, *P*. *megacephalus*, *P*. *rusticus* and *P*. *rohdei* (2.4–3.5 mm, combined values) by its smaller eye-nostril distance (1.7–2.3 mm) [8,9,37,38,39]. *Pithecopus araguaius* sp. n. is also diagnosed from *P*. *ayeaye*, *P*. *centralis*, *P*. *megacephalus*, *P*. *rohdei*, and *P*. *palliatus* (35–43.8 mm, combined values) by its smaller snout-vent length (28.3–34.4 mm) [8,9,37,38,39,40]. The new species also promptly distinguished from *P*. *rusticus* by lacking of slightly reticulated pattern on the palpebral membrane and throat region (pattern unique of *P*. *rusticus*) [39].

**Fig 5:**
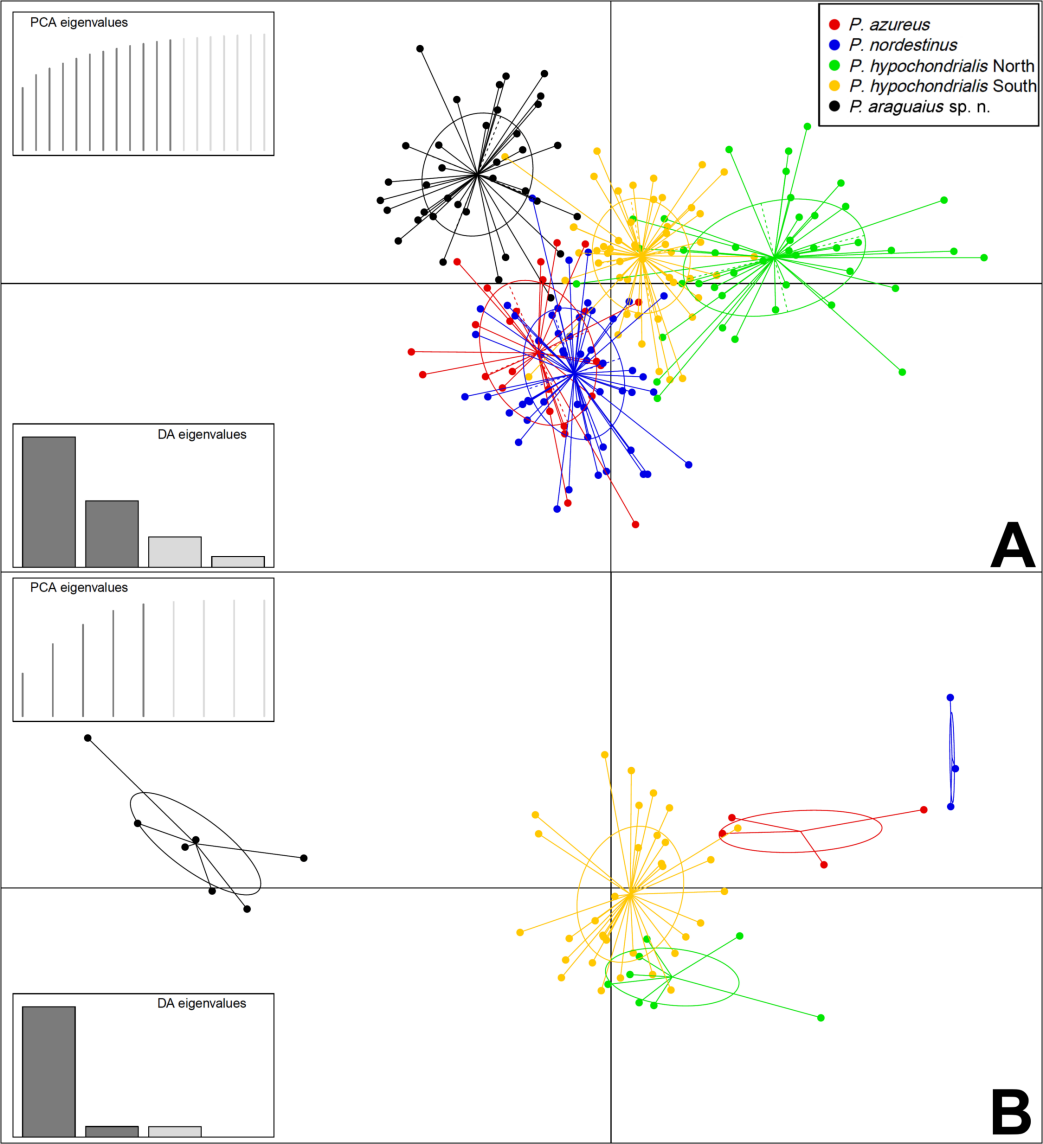
Scatterplot of the discriminant analyzes (DAPC) on the morphometric and acoustic datasets of *Pithecopus azureus*, *P*. *nordestinus*, *P*. *hypochondrialis* North, *P*. *hypochondrialis* South and *P*. *araguaius* sp. n.. (A) The two first axes on the morphometric data (12 first PCs, 95% retained variance). Variance explained by the axes: LD1 = 55% and LD2 = 28%. (B) The two first axes on the acoustic data (5 first PCs, 95% retained variance). LD1 = 86% and LD2 = 7%.

From its closer relatives (the lower land species), the new species is significantly smaller than *P*. *nordestinus*, *P*. *azureus* and *P*. *hypochondrialis “*North” and “South” in snout-vent length, head length, head width, axilla-groin length, eye-nostril distance, internarial distance, hand length, thigh length, tibia length and foot length (Exact Wilcoxon-Mann-Whitney Test: *p* < 0.01). The randomForest model on morphometric variables classified 35 individuals of *P*. *araguaius* sp. n. correctly (Table 3), only one male (the largest paratopotype AAG-UFU 3449; SVL = 33.8 mm) was misclassified. However, this same male is a call voucher specimen and was classified within new species group in acoustic multivariate analyzes (see results below). Accordingly, the DAPC discriminated the new species, with a greater separation along axis 1 (LD1 = 55%), but the axis 2 (LD2 = 28%) also contribute to separation. Tarsus (32%), tibia (17%), and thigh (14%) lengths mainly accounted for species separation along LD1 (Fig 5A); while eye-nostril (29%) and internarial (19%) distances, and head (10%) and hand (10%) lengths along LD2 (Fig 5A). The new species had the lowest values for all these variables along the axes 1 and 2, all Exact Wilcoxon-Mann-Whitney Tests with *p* < 0.01. The specimen AAG-UFU 3101 (SVL = 32.2 mm) of *P*. *hypochondrialis* South from Araguari (MG) is the smallest male for this group, due to this it was classified within new species cluster. Raw morphological measurement data of all examined specimens in S4 Table.

**Table 3:** Confusion matrix for the lowland species of the *Pithecopus* (except *P*. *palliatus*) based on the morphometric dataset by means of a Random Forests model.

	*P*. *araguaius* sp. n.	*P*. *azureus*	*P*. *hypochondrialis* North	*P*. *hypochondrialis* South	*P*. *nordestinus*	class.error
*P*. *araguaius* sp. n.	35	0	0	1	0	0.03
*P*. *azureus*	0	4	1	1	0	0.33
*P*. *hypochondrialis* North	0	0	34	8	1	0.21
*P*. *hypochondrialis* South	2	0	4	43	1	0.14
*P*. *nordestinus*	2	0	3	5	21	0.32

Settings: number of tree permutations = 1000; number of variables tried at each split = 4.0; error rate = 21%.

In comparison with the lowland species, the new species differs from *Pithecopus palliatus* by having a higher dominant frequency (2239–3316 vs. 1580 Hz) and by having a single note call, and not release as double notes as in *P*. *palliatus* [41]. The new species can be differentiated from *P*. *hypochondrialis* North and South, *P*. *azureus* and *P*. *nordestinus* by having a higher peak of dominant frequency (*p* <0.01, < 0.01, 0.01, and 0.01; respectively), a higher number of pulses per call (*p* <0.01, <0.01, 0.01, and 0.01), a longer core duration (*p* <0.01, <0.01, <0.01, and 0.01), and a higher number of pulses per core (*p* <0.01, <0.01, 0.01, and 0.01). From *P*. *hypochondrialis* North, the new species can also be differentiated by having a longer call duration (*p* < 0.01). In addition, *P*. *araguaius* sp. n. is differentiated from *P*. *hypochondrialis* South by its higher pulse rate (*p* < 0.01).

Regarding calls, the randomForest model resulted in total discrimination among *P*. *araguaius* sp. n. and *P*. *hypochondrialis* North and South/*P*. *azureus*/*P*. *nordestinus*, with all its individuals classified correctly (see Table 4). The DAPC also revealed substantial differentiation between the new species and *P*. *hypochondrialis* North and South/*P*. *azureus*/*P*. *nordestinus* (Fig 5B), with a greater separation along axis 1 (LD1 = 86%); while LD2 was only 7%. Pulses per core (31%), core duration (25%), isolated pulse (18%) and number of pulses per call (15%) mainly accounted for species separation along LD1 (Fig 5B). Isolated pulse (41%), call duration (35%), and number of pulses per call (11%) mainly accounted for species separation along LD2 (Fig 5B).

**Table 4:** Confusion matrix for lowland species of the *Pithecopus* (except *P*. *palliatus*) based on acoustic data by means of a Random Forests model.

	*P*. *araguaius* sp. n.	*P*. *azureus*	*P*. *hypochondrialis* North	*P*. *hypochondrialis* South	*P*. *nordestinus*	class.error
*P*. *araguaius* sp. n.	7	0	0	0	0	0.00
*P*. *azureus*	0	1	0	2	1	0.75
*P*. *hypochondrialis* North	0	0	6	2	0	0.25
*P*. *hypochondrialis* South	0	0	3	30	0	0.09
*P*. *nordestinus*	0	1	0	0	2	0.33

Settings: number of tree permutations = 1000; number of variables tried at each split = 3.0; error rate = 16%.

#### Description of the holotype

General aspect slender (Fig 2A and 2B); snout truncate in lateral (Fig 3A) and dorsal view (Fig 3B). Head wider than long; loreal region slightly concave; canthus rostralis rounded; nostrils small, subcanthal, placed latero-frontally, closer to snout tip than to eyes; internarial distance longer than eye-nostril distance and tympanum diameter, but smaller than eye diameter; eyes lateral-frontally positioned; tympanum nearly circular, with annuli undefined at the superior border; tympanum diameter less than half eye diameter; supratympanic dermal fold present, beginning on top of tympanum and ending nearly at the mouth corner; dorsolateral macrogland undistinguished; no vocal slits or external vocal sac; tongue nearly ovoid, free posteriorly, longer than wide; rudimentary vomerine teeth present, bordering choanae internally; choanae small, located laterally, slight rounded.

Upper arm thin and forearm robust; no finger webbing; comparative finger length when adpressed I<II<IV<III; finger discs poorly developed, smaller than the tympanum diameter; finger I enlarged at the base; nuptial asperity covering most of the dorsal surface of finger I, except the tip; palmar tubercles poorly developed (Fig 3C), subarticular tubercles developed, rounded and distinct from the supernumerary ones; inner and outer metacarpal tubercles undifferentiated; comparative toe length when adpressed II<III<I<V<IV; no toe webbing; plantar callosities poorly developed (Fig 3D), inner and outer metatarsal tubercles undifferentiated; subarticular tubercle developed, single and rounded; supernumerary tubercles rounded and poorly developed; legs slender, thigh slightly longer than tibia; heel reaches the posterior border of tympanum when a leg is adpressed to body; dorsal skin smooth; ventral skin granulated on belly and throat and smooth on thigh and foot. Left thigh slightly damaged ventrally due to tissue sampling.

#### Measurements of holotype (mm, abbreviations as in M&M)

SVL 30.6, HL 6.6, HW 9.6, AGL 14.6, ED 3.9, TD 1.6, END 2.1, IND 2.8, UAL 6.1, FAL 6.8, HAL 7.2, THL 12.9, TL 12.6, TAL 8.4, FL 10.1, 3FD 0.8, 4FD 0.8, 4TD 0.8, 5TD 0.9.

#### Color in life (n = 17 male paratopotypes)

Dorsal surfaces of forearm, tibia, tarsus and portions of foot and hand green (Fig 4A and 4B). A green stripe on dorsal surface of the thigh, which never reaches the final proximal thigh portion (Fig 4). Vertical dark stripe on flanks, dorsal surface of arm, medial surface of forearm and hidden part of the leg orange (Fig 4). Border of upper lip and upper eyelid with a white line that, on lip, never reaches the border of lower eyelid. Thin dark lines surrounding the upper surface of the fingers and toes (Fig 4C and 4D). In preservative the green areas become pale bluish gray and the orange parts become light cream or whitish and the dark stripes, upper lip and upper eyelid lines remain unchanged (Figs 2 and 3). All type specimens have a thin white line at the edge of the upper lip which reaches the border of lower eyelid and the incomplete green strip in the dorsal face of the thigh (Fig 4).

#### Variation

Size variation in Table 1. The specimens AAG-UFU 3443–3447 and ZUEC 21659 have small white dots on back. The specimens AAG-UFU 3443–44, 3446–49, 4880 and ZUEC 21657–60 have a well-defined dark stripe on edge of jaw (angular region). The specimens AAG-UFU 3446, 3448, 4878 and 4882 each have a symmetrical small dark spot on lower eyelid, next to the posterior corner of the eye. The specimen ZUEC 21659 has dark spots on the vocal sac. The specimens AAG-UFU 3442–46, 3448–49, 4877, 4879, 4881–82 and ZUEC 21657–59 each have a fine white line in the border of upper eyelid surrounded by a fine dark line. The specimens AAG-UFU 3442–48, 4877–78, 4880, 4882, ZUEC 21657–60 have a slightly darkened terminal discs.

#### Etymology

The epithet *araguaius* it is masculine latinized form of the indigenous Tupi word “araguaia*”*, a reference to the Araguaia River, which cross the type-locality of the new species.

#### Advertisement call

**Fig 6:**
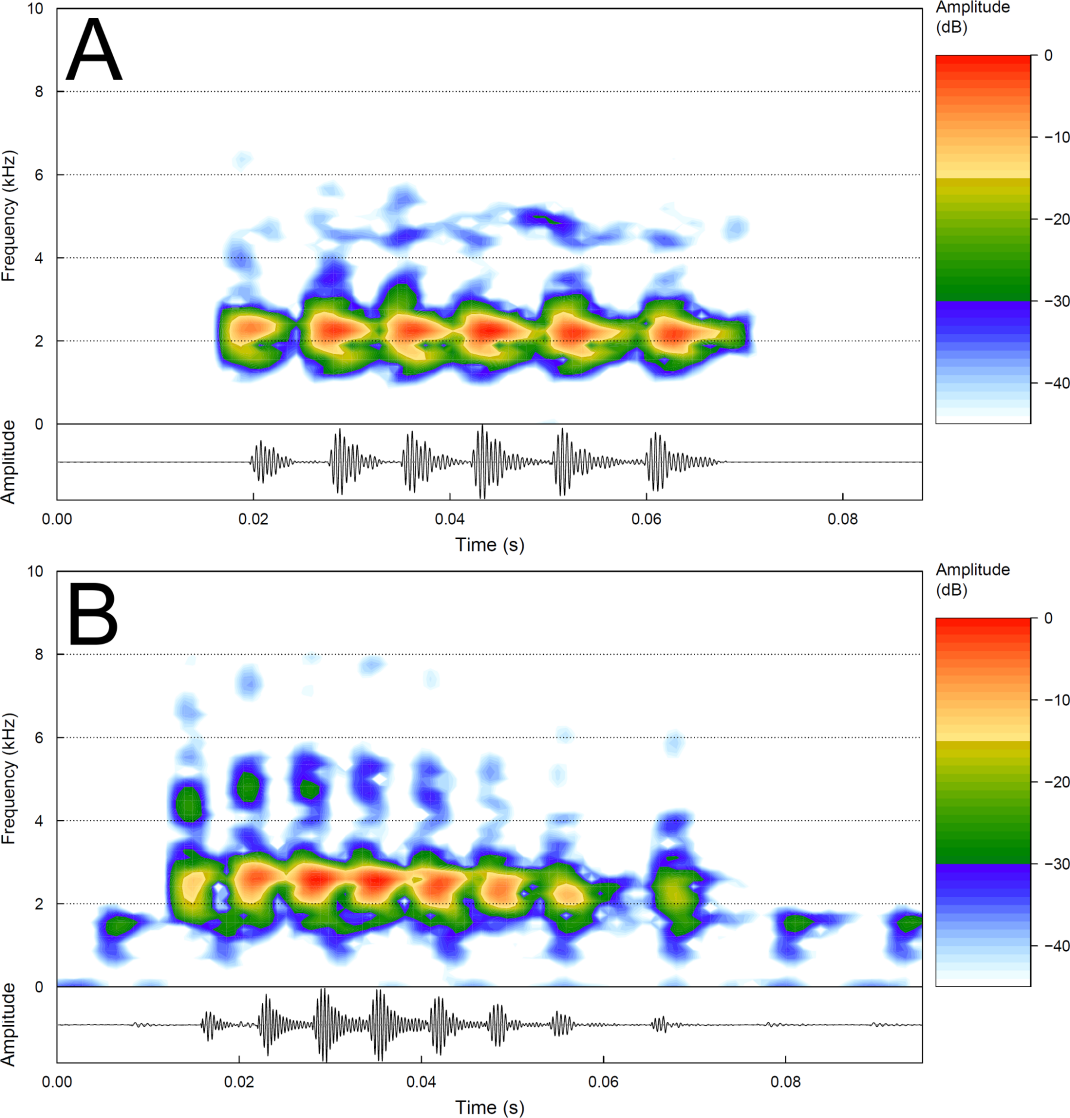
Audiospectrograms (above) and corresponding oscillograms (below) of the advertisement call of two topotypes of *Pithecopus araguaius* sp. n.. (A) Call core with six pulses and no isolated pulse, noticed in all 7 recorded males (22 calls = 41%); sound file: Pithec_araguaiusPontalAraguMT6aFSA_AAGb.wav; voucher (AAG-UFU 3449); (B) call core with seven pulses followed by one isolated pulse (around 0.07 s), noticed in only one male (2 calls = 4%); sound file: Pithec_araguaiusPontalAraguMT1cAAGmt.wav; voucher (AAG-UFU 4877). See S2 Table for further details of the sound files.

See also S1 Table (Appendix B) for further call traits definitions. Seven males of the new species were recorded, 54 calls analyzed. Quantitative traits are summarized in Table 2. The advertisement call of *Pithecopus araguaius* sp. n. consists of a single pulsed note emitted sporadically (Fig 6). Generally, the notes are composed of a long and strong group of pulses (core), and rarely by isolated weak final pulses. The core portion lasted 28–48 ms (mean = 39.0; SD = 5.0; n = 54) and have 5–8 pulses per core (mean = 6.0; SD = 0.5; n = 54). Also considering the isolated pulses, notes lasted 28–63 ms (mean = 41.0, SD = 4.9; n = 54) and have 5–8 pulses (mean = 6.0, SD = 0.6, n = 319). When present (only one male), isolated pulses were limited to one and lasted 2–5 ms (mean = 3.5; n = 5). The interval between the core and isolated pulses (Table 2) varied from 5–8 ms (mean = 6.0; n = 5). The pulse duration varied from 2–17 ms (mean = 5.1, SD = 1.3, n = 398), emitted at rates of 114–206 pulses/second (mean = 155.0, SD = 20.0, n = 54). The arrangement of pulses had the following patterns: (1) core with six pulses and no isolated pulse (41%; n = 22 calls, noticed in all 7 recorded males; see Fig 6A); (2) core with five pulses and no isolated pulse (33%; n = 18 calls, noticed in 4 males); (3) core with seven pulses and no isolated pulse (13%; n = 7 calls, in 4 males); (4) core with six pulses and one isolated pulse (6%; n = 3 calls, in 1 male); (5) core with seven pulses followed by one isolated pulse (see Fig 6B) and (6) core with eight pulses and no isolated pulse also was found and had the same frequency as other calls (4%; n = 2 calls, in 1 male). The peak of dominant frequency varied from 2240–3316 Hz (mean = 2540 Hz, SD = 308; n = 54).

**Fig 7:**
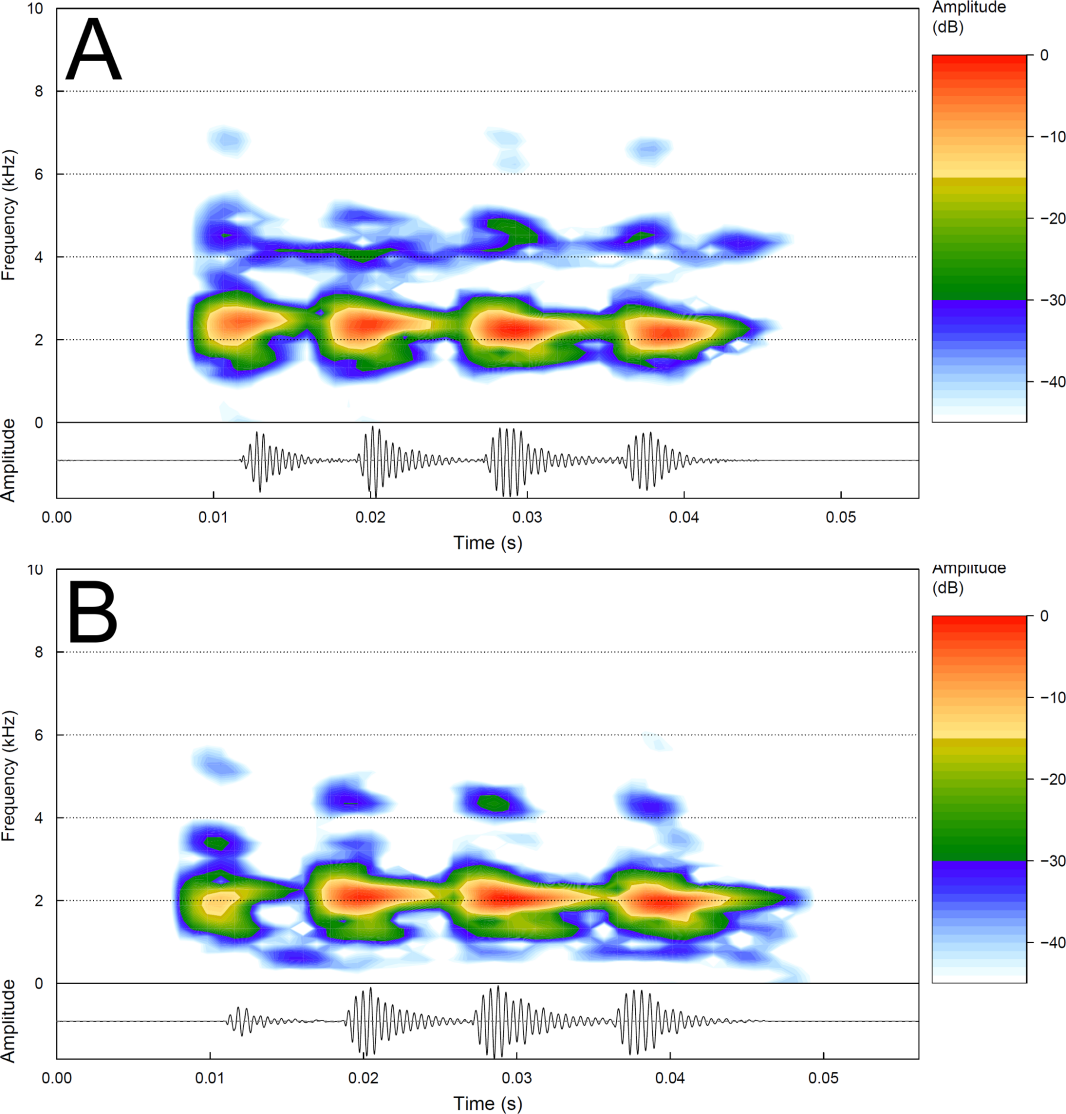
Audiospectrograms (above) and corresponding oscillograms (below) of the advertisement call of *Pithecopus hypochondrialis* South. (A) Call of a male from Barra do Garças (MT, Brazil), a locality less than 3 km from the type locality of the new species; sound file: Pithec_hypochBarraGarcasMT2bAAGmt.wav; voucher (AAG-UFU 1083). (B) A male from Uberlândia (MG); sound file: Pithec_hypochUberlMG9aAAGm671.wav; unvouchered recording. Note that both call cores have four pulses, this pattern was noticed in 95 calls (= 53%).

In our sample, the advertisement call of the sister species of the new taxon, *Pithecopus hypochondrialis* South (33 males, 180 calls), consists of a short note, with sporadic emission (quantitative call traits are summarized in Table 2; Fig 7). The core duration varied from 19–61 ms (mean = 33.2, SD = 3.2; n = 180) and the intervals between core and isolated pulses varied from 5–21 ms (mean = 12.5, SD = 3.4; n = 64). The number of pulses per core varied from 3–5 pulses (mean = 4.0, SD = 0.2; n = 180) (Fig 6); with intervals (or no interval) within core pulses from 1–7 ms (mean = 2.1, SD = 0.9; n = 539). Pulses were arranged in (1) core with four pulses and no isolated pulse (53%; n = 95 calls) (see Fig 7A and Fig 7B); (2) core with four pulses followed by an isolated pulse (31%; n = 55 calls); (3) core with five pulses and no isolated pulse (7%; n = 13 calls); (4) core with three pulses and no isolated pulse (4%; n = 8 calls); (5) core with three pulses followed by one isolated pulse (4%; n = 7 calls) and (6) core with five pulses followed by one isolated pulse (1%; n = 2 calls). The call duration lasted 22–72 ms (mean = 39.1, SD = 8.8; n = 180 calls), with 3–6 pulses (mean = 4.2, SD = 0.4, n = 719), which the duration varied from 2–15 ms (mean = 7.0 ms, SD = 1.1; n = 719), emitted at rates of 66–160 pulses/second (mean = 121.0, SD = 12.6; n = 719). The peak of dominant frequency varied from 1781–2625 (mean = 2170, SD = 145; n = 180).

#### Phylogenetic relationships

**Fig 8:**
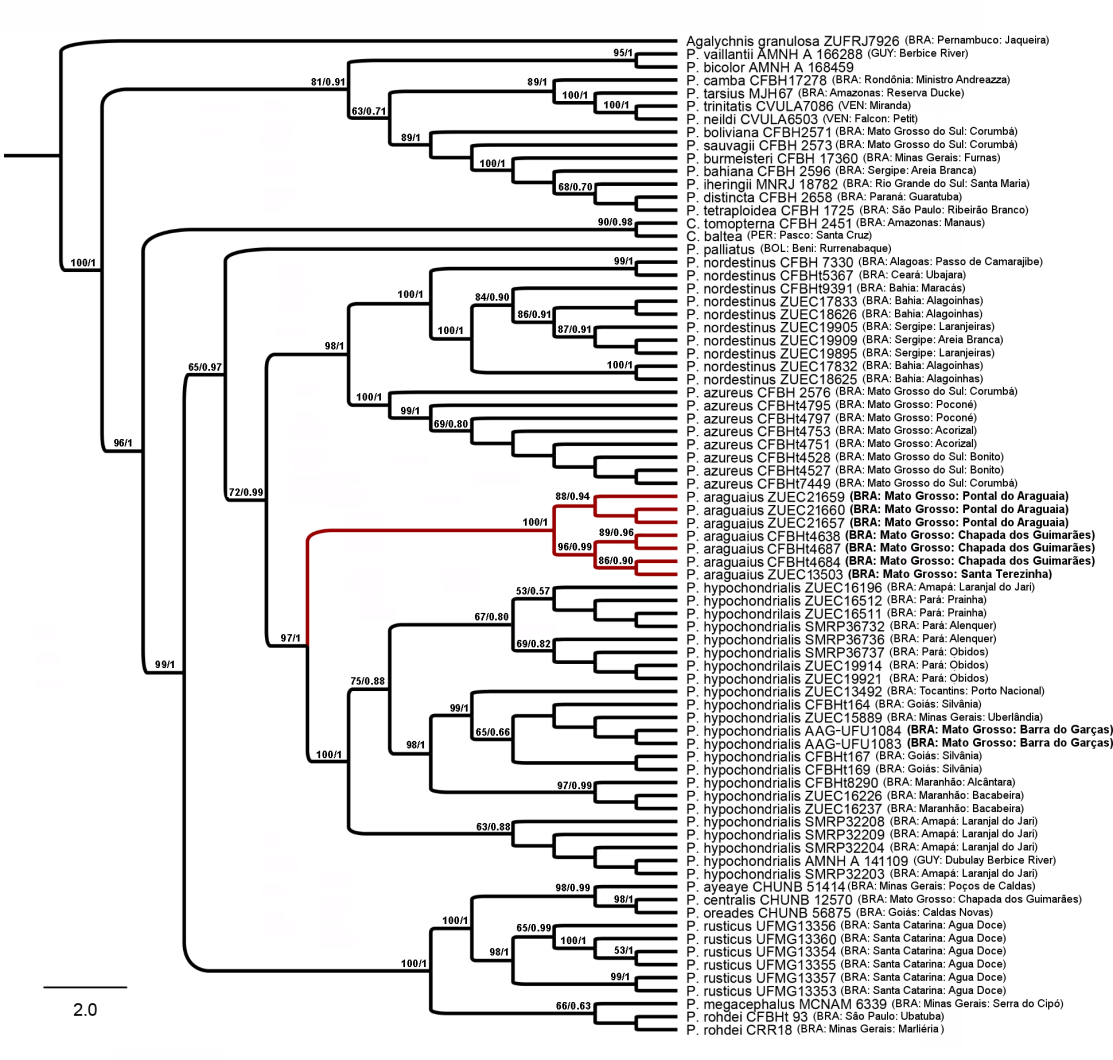
Phylogenetic relationships of the *Pithecopus* genus based in 1490 pb of the 16S rDNA mitochondrial genes. In the strict consensus cladogram inferred using the maximum parsimony (MP) criterion, implemented in the TNT program; the numbers above the branches show the bootstrap support and Bayesian posterior probabilities, respectively.

The topologies inferred by parsimony criterion and Bayesian inference were congruent one each other and similar to the phylogenetic relations recovered by Faivovich et al. [5], Bruschi et al. [7,39] and Duellman et al. [4]. In all our analyses, the paratopotypes of the *P*. *araguaius* sp. n. were nested with additional specimens from Chapada dos Guimarães and Santa Terezinha (both MT), forming a highly supported monophyletic group in both phylogenetics methods (Fig 8). This clade is the sister group of a clade composed by specimens of *P*. *hypochondrialis* North and South.

Specimens from the adjacent municipality of Barra do Garças (Fig 1) were recovered within *P*. *hypochondrialis* clade, nested with *P*. *hypochondrialis* South populations (specimens AAG-UFU 1083 and 1084; see topology in Fig 8). The genetic distances between *P*. *araguaius* sp. n. and other *Pithecopus* species ranged from 4 to 9% (Table 5); within the lowland species the genetic divergence levels varied from 4 to 7% (Table 5). Besides the high genetic distance (Table 5) and distinct phylogenetic position (Fig 8), morphometric and bioacoustics data of *P*. *hypochondrialis* from Barra do Garças are in our morphometric and acoustic multivariate analyses, and in both cases, they were not recovered within the clusters of the new species (Fig 5).

**Table 5:** Uncorrected pairwise distances between 16S mitochondrial sequences of the species of the *Pithecopus* genus.

Species	1	2	3	4	5	6	7	8	9	10	11	12
1- *Pithecopus araguaius* sp. n.	*-*											
2- *P*. *araguaius* sp. n. (Chapada dos Guimarães/MT)	0.01	-										
3- *P*. *hypochondrialis* (Barra dos Garças/MT)	**0.04**	0.04	-									
4- *P*. *hypochondrialis*	**0.04**	0.04	0.01	-								
5- *P*. *azureus*	**0.06**	0.05	0.05	0.05	-							
6- *P*. *nordestinus*	**0.07**	0.06	0.06	0.05	0.05	-						
7- *P*. *centralis*	**0.08**	0.07	0.08	0.08	0.08	0.08	-					
8- *P*. *oreades*	**0.08**	0.07	0.08	0.08	0.08	0.08	0.01	-				
9- *P*. *ayeaye*	**0.06**	0.05	0.08	0.07	0.07	0.07	0.03	0.03	-			
10- *P*. *rohdei*	**0.09**	0.08	0.07	0.07	0.07	0.08	0.04	0.04	0.06	-		
11- *P*. *rohdei_II*	**0.08**	0.08	0.07	0.07	0.07	0.09	0.04	0.04	0.05	0.04	-	
12- *P*. *megacephalus*	**0.08**	0.07	0.07	0.07	0.06	0.06	0.06	0.06	0.07	0.04	0.05	-
13- *P*. *rusticus*	**0.07**	0.06	0.06	0.07	0.07	0.08	0.05	0.05	0.05	0.04	0.05	0.04

#### Distribution

Based on the morphological and genetic similarities between populations from Pontal do Araguaia, Chapada dos Guimarães and Santa Terezinha (all MT) [7], the specific identity between all three is well supported. Chapada dos Guimarães and Santa Terezinha are about 380 km west and 630 km north from the type-locality of *P*. *araguaius* sp. n., respectively. Based on the distribution provided by Bruschi et al. [7], the range of the new species overlaps with that of *P*. *hypochondrialis* South, but not with those of *P*. *azureus* and *P*. *nordestinus* (see their Fig 2).

The genetic distance between the topotypes and specimens from Chapada dos Guimarães which are around 400 km apart shown low levels of divergence (*P*-distance 1%) in comparison with the *P-*distance of the 4% between individuals from Barra dos Garças (Table 5). The Mantel test scores (r = 0,083) (S2 File) formally reveled that geographic distances did not plays an important role in the distribution of genetic variation detected among samples and represent a strong evidence of the complete genetic isolation between *P*. *hypochondrialis* South and *P*. *araguaius* sp. n.. Monmonier algorithm detected biogeographic boundaries between populations from Pontal do Araguaia + Chapada dos Guimães *versus* Barra dos Garças that could be limited the gene flow among populations and represent putative origin of high genetic variation detected, however what are these biogeographical boundaries should be carefully evaluated in the future (S2 File).

## Discussion

A large amount of Brazilian Anuran hidden diversity has been revealed by the application of molecular genetics and its integration into taxonomic researches [39,42,43]. Phenotypic and genotypic evidences allowed the recognition of the new species here described. *Pithecopus araguaius* sp. n. have an evolutionary history related to the remaining lowland species of the Brazilian Cerrado and possibly has been historically confused to *P*. *hypochondrialis* due the taxonomic problems related to its definition and sympatry. Particularly, the inclusion of *P*. *hypochondrialis* samples from Barra dos Garças in our analyses was a crucial evidence to postulate *P*. *araguaius* sp. n. as a valid new species, as evidenced by the high level of genetic distance between them (noteworthy is that *P*. *hypochondrialis* AAG-UFU 1083 is a molecular, acoustic and morphometric voucher).

Indeed, the routine morphological identification of the *Pithecopus* species is not easy, mostly due to the high level of conservative morphology observed among *P*. *hypochondrialis*, *P*. *azureus* and *P*. *nordestinus*. Even to experienced herpetologists it has been difficult to separate these taxa when compared to the “nomimal species”, what has led to a repeated history of nomenclature changes in this group [8]. For example, the diagnostic characters proposed by Caramaschi [8] to distinguish *P*. *hypochondrialis* from *P*. *azureus* are too variable within and among populations [7] and in some cases has failed to allow its discrimination. Ours findings also re-emphase the notion that the species richness within lowland *Pithecopus* species was underestimated.

Haga et al. [10] pointed that the similarities observed among the advertisement calls of the *P*. *hypochondrialis*, *P*. *azureus* and *P*. *nordestinus*, and the resulting difficulty in discriminating them, indicates that acoustic characters are uninformative for these closely related species. However, these same authors emphasized that molecular and cytogenetic datasets supported their independent specific identities, as suggested by Bruschi et al. [7]. Our results corroborated those highlighted by Haga et al. [10], once *Pithecopus araguaius* sp. n. was the only species with complete discrimination in the randomForest model and DAPC on acoustic dataset. It is noteworthy that the *P*. *azureus* and *P*. *nordestinus* (sister species) are morphometrically indistinguishable from one another according to these same analyzes. Therefore, even though the acoustics and morphometric differences found in our statistical analyses may be deemed unable to fully diagnose the new species from the other three species abovementioned, due to the slight overlaps in their traits, they provide sufficient evidence to support our hypothesis that this lineage is evolving independently.

The Cerrado domain shown more than 200 documented anuran species with a high level of endemism [44]. The biologists are fascinated for the questions about the mechanisms that has been promote this species diversity, and a set of evidences postulated about the strong influence of the local environmental gradients to drive the distribution pattern, and for consequence, the diversification of the species [1,45]. Thus, the recognition of the *P*. *araguaius* sp. n. is important to the knowledge of the frog richness and diversification pattern that operated in this region. Future phylogeographic studies would be valuable to resolve the evolutionary history of the lowland *Pithecopus* species group and the Cerrado domain role in this context.

## Supporting information

S1 File **Appendix A**. **Examined specimens of *Pithecopus* species**.
(DOC)

S2 File Mantel test and result of Monmonier algorithm. (DOC)

S1 Table **Appendix B**. **Acoustic terminology employed for the species of *Pithecopus***.
(DOC)

S2 Table **Appendix C**. Analyzed sound files (*.wav format) of the four *Pithecopus species*: *P*. *araguaius* sp. n., *P*. *hypochondrialis*, *P*. *nordestinus* and *P*. *azureus*.
All files deposited at the AAG sound collection (Universidade Federal de Uberlândia, Brazil) or at the Fonoteca Neotropical Jacques Vielliard (FNJV) (Universidade Estadual de Campinas, Brazil).
(DOC)

S3 Table GenBank details: Species, voucher number, sample locality, accession number and authors) of the sequences used for phylogenetic inferences. (DOC)

S4 Table Raw morphological measurement data (values in millimeters) of examined Pithecopus specimens which were used for statistical analysis. Abbreviation to collections: ZUEC (Museu de Zoologia da Unicamp, Universidade Estadual de Campinas, Brazil); AAG-UFU (Collection of frogs of the Museu de Biodiversidade do Cerrado, Universidade Federal de Uberlândia, Brazil); MNRJ (Museu Nacional do Rio de Janeiro, Universidade Federal do Rio de Janeiro, Brazil); CFBH (Célio F. B. Haddad, Universidade Estadual Paulista, Brazil). Morphometric traits: snout-vent length (SVL), hand length (HAL), forearm length (FAL), thigh length (THL), foot length (FL), head length (HL), head width (HW), eye diameter (ED), internarial distance (IND), tibia length (TL) (= shank length), tympanum diameter (TD), and eye-nostril distance (END), upper arm length (UAL), tarsus length (TAL), the disc diameters of third finger (3FD), fourth finger (4FD), fourth toe (4TD), fifth toe (5TD), and axilla-groin length (AGL).
(DOC)
