# Supplementary material for: Unrevealing the leaf frogs Cerrado diversity: A new species of Pithecopus (Anura, Arboranae, Phyllomedusidae) from the Mato Grosso state, Brazil
Source: PLoS One. 2017 Sep 27;12(9):e0184631. doi: 10.1371/journal.pone.0184631 (PMC5617161; doi:10.1371/journal.pone.0184631)
Supplement: S1 File — Appendix A. Examined specimens of Pithecopus species. (DOC) [file pone.0184631.s001.doc]

**S1 File**

**Appendix A.** Examined specimens of *Pithecopus* species

*Pithecopus araguaius* sp. n.–BRAZIL: MATO GROSSO: Pontal do Araguaia (AAG-UFU 3442–3449, 4877–4882, ZUEC 21657–21660); Chapada dos Guimarães (ZUEC 15884–88, 21644–45, 21647; CFBH 14321–22, 14367–70, 14406); Santa Terezinha (ZUEC 7457–7458, 13503). *Pithecopus azureus*–PARAGUAY: Asunción (MNRJ 13657–62; 13664–70); ARGENTINA: Corrientes (MNRJ 39995); BRAZIL: MATO GROSSO DO SUL: Bela Vista (AAG-UFU 0148–53; MNRJ 61567–71). *Pithecopus hypochondrialis*–BRAZIL: PARÁ: Belterra (ZUEC 16605, 16504–09); Prainha (ZUEC 16511, 16561, 16520, 16515, 16529–30); Monte Alegre (ZUEC 19916, 19938, 19940, 19944); Alenquer (ZUEC 19917, 19920, 19923, 19927, 19930, 19932); Oriximiná (ZUEC 19918, 19922, 19926, 19928); Óbidos (ZUEC 19921,19933, 19943, 19945). AMAPÁ: Laranjal do Jari: (ZUEC 16550–51, 16553, 16555, 16559, 16562); Serra do Navio: (AAG-UFU 5987–89, 5998–6000); MATO GROSSO: Barra do Garças (ZUEC 21650, AAG-UFU 3489–94,1078–84); GOIÁS: Guarani de Goiás (AAG-UFU 1963–64); Uruaçu (AAG-UFU 0991–93, 0996–99); Pirenópolis (AAG-UFU 0331, 0334); Padre Bernardo (AAG-UFU 0117–18); Chapada dos Veadeiros (AAG-UFU 1333); Mineiro (AAG-UFU 3410); TOCANTINS: Paranã (AAG-UFU 2827–30); Palmas (AAG-UFU 2779, 2817); MINAS GERAIS: Uberlândia (AAG-UFU 2311, 2313, 2315, 2299, 3256); Araguari (AAG-UFU 3101, 3116, 4573, 4689, 4936–37, 4832, 4834); Ituiutaba (AAG-UFU 0455, 1276); *Pithecopus nordestinus*–BRAZIL: BAHIA: Alagoinhas (ZUEC 18623–26); Maracás (MNRJ 13598–611, 35223–28, 60097); SERGIPE: Areia Branca (ZUEC 19882–85, 19887–94, 19898–99, 19901–02, 19906–07, 19909, 19911); Laranjeiras (ZUEC 19895, 19897, 19900, 19903, 19908,19912, 19913). *Pithecopus rohdei*–BRAZIL: RIO DE JANEIRO: Itaguaí (ZUEC 1223–24, 5229–30, 7716); Seropédica (ZUEC 16130). *Pithecopus megacephalus*–BRAZIL: MINAS GERAIS: Jaboticatubas (ZUEC 1651, 3004–06, 3428, 15444). *Pithecopus ayeaye*–BRAZIL: MINAS GERAIS: Poços de Caldas (AAG-UFU 1661–64, 3523–25; ZUEC 4160–1, 4289–91, 4293, 4470, 4481, 6854); Alpinópolis (AAG-UFU 0962–0964). *Pithecopus palliatus*–BRAZIL: ACRE: Cruzeiro do Sul (ZUEC 5388, 5392), Xapuri (ZUEC 5685–95, 5740–1, 5754, 5760). *Pithecopus rusticus*–BRAZIL: SANTA CATARINA: Água Doce (UFMG 13360–13362, 13353–13359).
