# Supplementary material for: Unrevealing the leaf frogs Cerrado diversity: A new species of Pithecopus (Anura, Arboranae, Phyllomedusidae) from the Mato Grosso state, Brazil
Source: PLoS One. 2017 Sep 27;12(9):e0184631. doi: 10.1371/journal.pone.0184631 (PMC5617161; doi:10.1371/journal.pone.0184631)

**S2 File**

**Mantel test and result of Monmonier algorithm.**

To aim the evaluated if geographic distances could be explain the genetic differences among populations from Pontal do Araguaia + Chapada dos Guimarães and Barra dos Garças, all in Mato Grosso states and exclude the potential effect of the genetic isolation by distances in ours results, we test these three populations from Mato Grosso by Mantel test. Additionally, we also test these samples under Monmonier algorithm to identifies homogeneous groups in the distribution from the samples and allows the associated of sequences groups with potential existing biogeographical boundaries. Ours Mantel test results (**A**) exclude the effect of the geographical distances as source of the strong genetic variation evidenced and biogeographic boundaries determined by Monmonier algorithm (**B**) reveals putative existing of local gene flow barrier among Pontal do Araguaia + Chapada dos Guimarães and Barra dos Garças. Note the polygon drawn in the distribution map, whose number of vertices is equal to the number of points tested and the barrier drawn by the program (red line) reveals between which groups of samples potentially exist a biogeographic barrier.


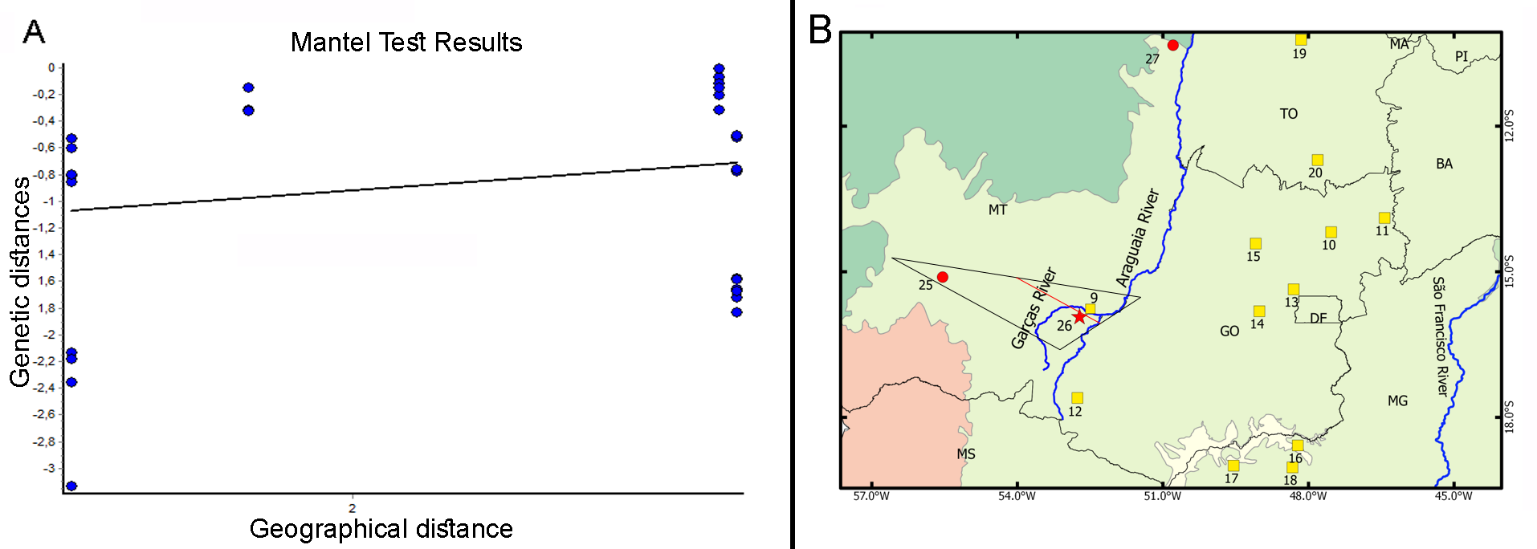

Supplement: S2 File — (DOC) [file pone.0184631.s002.doc]
