# Supplementary material for: Unrevealing the leaf frogs Cerrado diversity: A new species of Pithecopus (Anura, Arboranae, Phyllomedusidae) from the Mato Grosso state, Brazil
Source: PLoS One. 2017 Sep 27;12(9):e0184631. doi: 10.1371/journal.pone.0184631 (PMC5617161; doi:10.1371/journal.pone.0184631)
Supplement: S1 Table — Appendix B. Acoustic terminology employed for the species of Pithecopus. (DOC) [file pone.0184631.s003.doc]

**S1 Table**

**Appendix B. Acoustic terminology employed here for the species of *Pithecopus*.**

| **Acoustic traits** | **Definition** |
| --- | --- |
| Call duration (ms) | Time from beginning to end of one call, including isolated pulses; |
| Intercall interval (ms) | Time from the end of one call to beginning of the next |
| Calls rate per minute | Numbers of calls recorded multiplied by 60s divided by time total (from begin to first call recorded into the end the last call recorded) |
| Pulses/call | Number of pulses in the entire call. |
| Pulse duration (ms) | Mean duration of all pulses in core. Ranges given from original data and not by individual average. |
| Inter-pulse interval within core (ms) | Mean interval between core pulses. Ranges given from original data and not by individual average). |
| Core duration (ms) | Time from beginning to end of one core. = Isolated pulses, if present, disregarded. |
| Pulses/core | Number of pulses into the core. |
| Duration of isolated pulses (ms) | Mean of duration of the isolated pulses. Range, which was given from original data and not by individual average). |
| Number of isolated pulses | Number of isolated pulses in the calls (summed when in more than a group). |
| Interval between core and isolated pulse (ms) | Time from end to core to the first isolated pulse. |
| Pulse rate per second | Pulses per core/core duration |
| Minimum of dom. freq. (Hz) | Minimum frequency within the most energetic call band. |
| Maximum of dom. freq. (Hz) | Maximum frequency within the most energetic call band. |
| Peak of dominant frequency | Frequency with greatest energy within the most energetic call band (function Peak Frequency of Raven) |
| Fundamental frequency | Coincident to dominant frequency. |
