# Supplementary material for: Unrevealing the leaf frogs Cerrado diversity: A new species of Pithecopus (Anura, Arboranae, Phyllomedusidae) from the Mato Grosso state, Brazil
Source: PLoS One. 2017 Sep 27;12(9):e0184631. doi: 10.1371/journal.pone.0184631 (PMC5617161; doi:10.1371/journal.pone.0184631)
Supplement: S2 Table — Appendix C. Analyzed sound files (*.wav format) of the four Pithecopus species: P. araguaius sp. n., P. hypochondrialis, P. nordestinus and P. azureus. All files deposited at the AAG sound collection (Universidade Federal de Uberlândia, Brazil) or at the Fonoteca Neotropical Jacques Vielliard (FNJV) (Universidade Estadual de Campinas, Brazil). (DOC) [file pone.0184631.s004.doc]

**S2 Table**

**Appendix C.** **Analyzed sound files (*.wav format) of the four *Pithecopus* species: *P. araguaius* sp. n., *P. hypochondrialis*, *P. nordestinus* and *P. azureus*.**

| Label | Date | Municipality (state) | Time | Air (°C) | Water (°C) | Voucher  AAG-UFU |
| --- | --- | --- | --- | --- | --- | --- |
| Pithec_hypochAraguariMG2aTRC_LMmt.wav | 08 December 2009 | Araguari (MG) | 21:34 | 21.2 | 22.6 | - |
| Pithec_hypochAraguariMG3aTRC_LMmt.wav | 08 December 2009 | Araguari (MG) | 21:54 | 21.2 | 22.6 | - |
| Pithec_hypochAraguariMG4aTRC_LMmt.wav | 08 December 2009 | Araguari (MG) | 22:13 | 21.2 | 22.6 | - |
| Pithec_hypochAraguariMG5aTRC_LMmt.wav | 08 December 2009 | Araguari (MG) | 22:13 | 21.2 | 22.6 | 4832 |
| Pithec_hypochAraguariMG6aTRC_LMmt.wav | 09 December 2009 | Araguari (MG) | 00:30 | 20.0 | 22.6 | - |
| Pithec_hypochAraguariMG7aTRC_LMmt.wav | 09 December 2009 | Araguari (MG) | 00:26 | 20.0 | 22.6 | - |
| Pithec_hypochAraguariMG8aTRC_LMmt.wav | 09 December 2009 | Araguari (MG) | 01:09 | 20.0 | 22.6 | - |
| Pithec_hypochAraguariMG9aAAGm.wav | 06 November 2010 | Araguari (MG) | 02:10 | 22.0 | 23.0 | - |
| Pithec_hypochAraguariMG10aAAGm.wav | 06 November 2010 | Araguari (MG) | 22:35 | 22.5 | 23.0 | - |
| Pithec_hypochBarraGarcasMT1aAAGm.wav | 20 February 2012 | Barra do Garças (MT) | 00:19 | 22.0 | 25.0 | 1082 |
| Pithec_hypochBarraGarcasMT2aAAGmt.wav | 19 February 2012 | Barra do Garças (MT) | 23:34 | 22.0 | 25.0 | 1083 |
| Pithec_hypochBarraGarcasMT2bAAGmt.wav | 19 February 2012 | Barra do Garças (MT) | 23:39 | 22.0 | 25.0 | 1083 |
| Pithec_hypochBarraGarcasMT2cAAGmt.wav | 19 February 2012 | Barra do Garças (MT) | 23:42 | 22.0 | 25.0 | 1083 |
| Pithec_hypochBarraGarcasMT3aAAGm671.wav | 09 January 2014 | Barra do Garças (MT) | 22:31 | 26.0 | 29.0 | - |
| Pithec_hypochBarraGarcasMT4aAAGm671.wav | 09 January 2014 | Barra do Garças (MT) | 22:31 | 26.0 | 29.0 | 3489 |
| Pithec_hypochBarraGarcasMT4bAAGm671.wav | 09 January 2014 | Barra do Garças (MT) | 22:35 | 26.0 | 29.0 | 3489 |
| Pithec_hypochBarraGarcasMT5aCSB_AAGm671.wav | 09 January 2014 | Barra do Garças (MT) | 22:42 | 26.0 | 29.0 | - |
| Pithec_hypochBrasiliaDF1aAAGm671.wav | 13 January 2015 | Brasília (DF) | 22:33 | 24.0 | 25.0 | - |
| Pithec_hypochBrasiliaDF1bAAGm671.wav | 13 January 2015 | Brasília (DF) | 22:33 | 24.0 | 25.0 | - |
| Pithec_hypochBrasiliaDF1cAAGm671.wav | 13 January 2015 | Brasília (DF) | 22:49 | 24.0 | 25.0 | - |
| Pithec_hypochPadBernarGO1aAAGm.wav | 10 December 2010 | Padre Bernardo (GO) | 23:00 | 24.6 | 26.5 | - |
| Pithec_hypochPadBernarGO1bAAGm.wav | 10 December 2010 | Padre Bernardo (GO) | 23:07 | 24.6 | 26.5 | - |
| Pithec_hypochPirenopGO1aAAGm.wav | 11 February 2011 | Pirenópolis (GO) | 20:13 | 23.0 | 27.0 | 0331 |
| Pithec_hypochPirenopGO2aAAGm.wav | 12 February 2011 | Pirenópolis (GO) | 22:00 | 24.0 | 26.0 | 0332 |
| Pithec_hypochPirenopGO3aAAGm.wav | 12 February 2011 | Pirenópolis (GO) | 22:14 | 24.0 | 26.0 | 0333 |
| Pithec_hypochUberlMG1aAAGm.wav | 22 December 2009 | Uberlândia (MG) | 03:00 | 18.0 | 20.0 | - |
| Pithec_hypochUberlMG3aAAGm.wav | 22 December 2009 | Uberlândia (MG) | 03:40 | 18.0 | 20.0 | - |
| Pithec_hypochUberlMG4aAAGm.wav | 22 December 2009 | Uberlândia (MG) | 03:41 | 18.0 | 20.0 | - |
| Pithec_hypochUberlMG7aAAGm671.wav | 14 October 2011 | Uberlândia (MG) | 20:10 | 21.0 | 25.0 | - |
| Pithec_hypochUberlMG8bAAGm671.wav | 14 October 2011 | Uberlândia (MG) | 20:05 | 21.0 | 25.0 | - |
| Pithec_hypochUberlMG9aAAGm671.wav | 14 October 2011 | Uberlândia (MG) | - | 21.0 | 25.0 | - |
| Pithec_hypochUberlMG10aAAGm671.wav | 14 October 2011 | Uberlândia (MG) | 20:23 | 21.0 | 25.0 | - |
| Pithec_hypochUruacuGO1aAAGm.wav | 25 January 2012 | Uruaçu (GO) | 22:31 | 23.0 | 27.0 | 0991 |
| Pithec_hypochUruacuGO1bAAGm.wav | 25 January 2012 | Uruaçu (GO) | 22:41 | 23.0 | 27.0 | 0991 |
| Pithec_hypochUruacuGO2aAAGm.wav | 25 January 2012 | Uruaçu (GO) | 22:54 | 23.0 | 27.0 | 0992 |
| Pithec_hypochUruacuGO2bAAGm.wav | 25 January 2012 | Uruaçu (GO) | 23:13 | 23.0 | 27.0 | 0992 |
| Pithec_hypochUruacuGO3aAAGm.wav | 25 January 2012 | Uruaçu (GO) | 23:15 | 23.0 | 27.0 | 0993 |
| Pithec_hypochUruacuGO5aAAGm.wav | 25 January 2012 | Uruaçu (GO) | 23:25 | 23.0 | 27.0 | - |
| Pithec_hypochUruacuGO6aAAGm.wav | 25 January 2012 | Uruaçu (GO) | 23:34 | 23.0 | 27.0 | - |
| Pithec_hypochUruacuGO6bAAGm.wav | 25 January 2012 | Uruaçu (GO) | 23:38 | 23.0 | 27.0 | - |
| Pithec_araguaiusPontalAraguMT1aAAGmt.wav | 15 February 2010 | Pontal do Araguaia (MT) | 22:20 | 22.0 | 26.0 | 4877 |
| Pithec_araguaiusPontalAraguMT1bAAGmt.wav | 15 February 2010 | Pontal do Araguaia (MT) | 22:25 | 22.0 | 26.0 | 4877 |
| Pithec_araguaiusPontalAraguMT1cAAGmt.wav | 15 February 2010 | Pontal do Araguaia (MT) | 22:30 | 22.0 | 26.0 | 4877 |
| Pithec_araguaiusPontalAraguMT2aAAGmt.wav | 15 February 2010 | Pontal do Araguaia (MT) | 22:55 | 22.0 | 26.0 | 4878 |
| Pithec_araguaiusPontalAraguMT2bAAGmt.wav | 15 February 2010 | Pontal do Araguaia (MT) | 22:57 | 22.0 | 26.0 | 4878 |
| Pithec_araguaiusPontalAraguMT2cAAGmt.wav | 15 February 2010 | Pontal do Araguaia (MT) | 23:00 | 22.0 | 26.0 | 4878 |
| Pithec_araguaiusPontalAraguMT2dAAGmt.wav | 15 February 2010 | Pontal do Araguaia (MT) | 23:05 | 22.0 | 26.0 | 4878 |
| Pithec_araguaiusPontalAraguMT3aAAGm671.wav | 06 January 2014 | Pontal do Araguaia (MT) | 20:41 | 26.0 | 30.0 | 3444 |
| Pithec_araguaiusPontalAraguMT3bAAGm671.wav | 06 January 2014 | Pontal do Araguaia (MT) | 20:43 | 26.0 | 30.0 | 3444 |
| Pithec_araguaiusPontalAraguMT3cAAGm671.wav | 06 January 2014 | Pontal do Araguaia (MT) | 20:45 | 26.0 | 30.0 | 3444 |
| Pithec_araguaiusPontalAraguMT5aIAH_AAGmt.wav | 07 January 2014 | Pontal do Araguaia (MT) | 23:46 | 25.0 | 31.0 | - |
| Pithec_araguaiusPontalAraguMT5bIAH_AAGmt.wav | 07 January 2014 | Pontal do Araguaia (MT) | 23:56 | 25.0 | 31.0 | - |
| Pithec_araguaiusPontalAraguMT5cIAH_AAGmt.wav | 07 January 2014 | Pontal do Araguaia (MT) | 00:09 | 25.0 | 31.0 | - |
| Pithec_araguaiusPontalAraguMT5dIAH_AAGmt.wav | 07 January 2014 | Pontal do Araguaia (MT) | 00:09 | 25.0 | 31.0 | - |
| Pithec_araguaiusPontalAraguMT5eIAH_AAGmt.wav | 07 January 2014 | Pontal do Araguaia (MT) | 00:11 | 25.0 | 31.0 | - |
| Pithec_araguaiusPontalAraguMT6aFSA_AAGb.wav | 07 January 2014 | Pontal do Araguaia (MT) | - | 25.0 | 31.0 | 3449 |
| Pithec_araguaiusPontalAraguMT7aAAGm671.wav | 01 December 2014 | Pontal do Araguaia (MT) | 21:05 | 26.0 | 27.0 | - |
| Pithec_araguaiusPontalAraguMT9aAAGm671.wav | 03 December 2014 | Pontal do Araguaia (MT) | 02:34 | 26.0 | 27.0 | 5042 |
| Pithec_hypochondSNavioAP1aAAGm661MK2.wav | 27 March 2017 | Serra do Navio (AP) | 19:48 | 26.0 | - | - |
| Pithec_hypochondSNavioAP2aAAGm661MK2.wav | 27 March 2017 | Serra do Navio (AP) | 22:11 | 26.0 | - | - |
| Pithec_hypochondSNavioAP2bAAGm661MK2.wav | 27 March 2017 | Serra do Navio (AP) | 22:31 | 26.0 | - | - |
| Pithec_hypochondSNavioAP4aAAGm661MK2.wav | 27 March 2017 | Serra do Navio (AP) | 22:57 | 26.0 | - | - |
| Pithec_hypochondSNavioAP5aAAGm661MK2.wav | 28 March 2017 | Serra do Navio (AP) | 19:24 | 26.0 | - | 5998 |
| Pithec_hypochondSNavioAP6aAAGm661MK2.wav | 28 March 2017 | Serra do Navio (AP) | 20:21 | 26.0 | - | - |
| Pithec_hypochondSNavioAP7aAAGm661MK2.wav | 28 March 2017 | Serra do Navio (AP) | 20:34 | 26.0 | - | - |
| Pithec_hypochondSNavioAP7bAAGm661MK2.wav | 28 March 2017 | Serra do Navio (AP) | 20:55 | 26.0 | - | - |
| Pithec_hypochondSNavioAP7cAAGm661MK2.wav | 28 March 2017 | Serra do Navio (AP) | 20:57 | 26.0 | - | - |
| Pithec_hypochondSNavioAP7dAAGm661MK2.wav | 28 March 2017 | Serra do Navio (AP) | 20:58 | 26.0 | - | - |
| Pithec_hypochondSNavioAP7eAAGm661MK2.wav | 28 March 2017 | Serra do Navio (AP) | 20:59 | 26.0 | - | - |
| Pithec_hypochondSNavioAP8aAAGm661MK2.wav | 29 March 2017 | Serra do Navio (AP) | 20:37 | 26.0 | - | - |
| Pithec_hypochondSNavioAP9aAAGm661MK2.wav | 29 March 2017 | Serra do Navio (AP) | 20:39 | 26.0 | - | - |
| Pithec_hypochondSNavioAP9bAAGm661MK2.wav | 29 March 2017 | Serra do Navio (AP) | 20:43 | 26.0 | - | - |
| Pithec_azurBelaVistaMS1aTRC_AAGmt.wav | 18 December 2010 | Bela Vista (MS) | 20:40 | 26.0 | 32.0 | 0148 |
| Pithec_azurBelaVistaMS1bTRC_AAGmt.wav | 18 December 2010 | Bela Vista (MS) | 20:43 | 26.0 | 32.0 | 0148 |
| Pithec_azurBelaVistaMS1cTRC_AAGmt.wav | 18 December 2010 | Bela Vista (MS) | 20:44 | 26.0 | 32.0 | 0148 |
| Pithec_azurBelaVistaMS2aTRC_AAGmt.wav | 18 December 2010 | Bela Vista (MS) | 20:52 | 26.0 | 32.0 | 0149 |
| Pithec_azurBelaVistaMS2bTRC_AAGmt.wav | 18 December 2010 | Bela Vista (MS) | 20:55 | 26.0 | 32.0 | 0149 |
| Pithec_azurBelaVistaMS2cTRC_AAGmt.wav | 18 December 2010 | Bela Vista (MS) | 21:01 | 26.0 | 32.0 | 0149 |
| Pithec_azurBelaVistaMS2dTRC_AAGmt.wav | 18 December 2010 | Bela Vista (MS) | - | 26.0 | 32.0 | 0149 |
| Pithec_azurBelaVistaMS3aTRC_AAGmt.wav | 18 December 2010 | Bela Vista (MS) | 21:06 | 26.0 | 32.0 | 0150 |
| Pithec_azurBelaVistaMS4aTRC_AAGmt.wav | 18 December 2010 | Bela Vista (MS) | 23:14 | 31.0 | 27.0 | 0151 |
| FNJV_0012243_AreiaBranca_SE.wav | 05 May 2011 | Areia Branca (SE) | 21:20 | 27.0 | - | - |
| FNJV_0012244_AreiaBranca_SE.wav | 05 May 2011 | Areia Branca (SE) | 21:22 | 27.0 | - | - |
| FNJV_0012245_AreiaBranca_SE.wav | 05 May 2011 | Areia Branca (SE) | 21:22 | 27.0 | - | - |
| FNJV_0012247_Muruim_SE.wav | 06 May 2011 | Muruim (SE) | 21:10 | 27.0 | - | - |
| FNJV_0032463_Itabaiana_SE.wav | 19 August 2006 | Itabaiana (SE) | 00:00 | 21.0 | - | - |

All files deposited at the AAG sound collection (Universidade Federal de Uberlândia, Brazil) or at the Fonoteca Neotropical Jacques Vielliard (FNJV) (Universidade Estadual de Campinas, Brazil).
