# Supplementary material for: Unrevealing the leaf frogs Cerrado diversity: A new species of Pithecopus (Anura, Arboranae, Phyllomedusidae) from the Mato Grosso state, Brazil
Source: PLoS One. 2017 Sep 27;12(9):e0184631. doi: 10.1371/journal.pone.0184631 (PMC5617161; doi:10.1371/journal.pone.0184631)
Supplement: S3 Table — (DOC) [file pone.0184631.s005.doc]

**S3 Table**

**GenBank details: species, voucher number, sample locality, accession number and authors) of the sequences used for phylogenetic inferences.**

| **Species** | **Voucher** | **Locality** | **rRNA16S** | **Reference** |
| --- | --- | --- | --- | --- |
| *Agalychnis granulosa* | ZUFRJ 7926 | Brazil, Pernambuco, Jaqueira | AY843687 | Faivovich et al. 2005 |
| *Callimedusa baltea* | To be deposited in SMRP | Peru, Pasco, Santa Cruz | GQ366252 | Faivovich et al. 2010 |
| *Callimedusa tomopterna* | CFBH 2451 | Brazil, Amazonas, Manaus | GQ366286 | Faivovich et al. 2010 |
| *Pithecopus ayeaye* | CHUNB 51414 | Brazil, Minas Gerais, Poços de Caldas | GQ366245 | Faivovich et al. 2010 |
| *Pithecopus azureus* | CFBH 2576 | Brazil, Mato Grosso do Sul, Corumbá | GQ366248 | Faivovich et al. 2010 |
| *Pithecopus azureus* | CFBHt 7449 | Brazil, Mato Grosso do Sul, Corumbá | MF926328 | Present study |
| *Pithecopus azureus* | CFBHt 4795 | Brazil, Mato Grosso, Poconé | MF926327 | Present study |
| *Pithecopus azureus* | CFBHt 4797 | Brazil, Mato Grosso, Poconé | MF926312 | Present study |
| *Pithecopus azureus* | CFBHt 4753 | Brazil, Mato Grosso, Acorizal | MF926326 | Present study |
| *Pithecopus azureus* | CFBHt 4751 | Brazil, Mato Grosso, Acorizal | MF926325 | Present study |
| *Pithecopus azureus* | CFBHt 4528 | Brazil, Mato Grosso do Sul, Bonito | MF926324 | Present study |
| *Pithecopus azureus* | CFBHt 4527 | Brazil, Mato Grosso do Sul, Bonito | MF926323 | Present study |
| *Phyllomedusa bahiana* | CFBH 2596 | Brazil, Sergipe, Areia Branca | GQ366251 | Faivovich et al. 2010 |
| *Phyllomedusa bicolor* | AMNH A-168459 | Pet trade (Germany) | AY843723 | Faivovich et al. 2005 |
| *Phyllomedusa boliviana* | CFBH 2571 | Brazil, Mato Grosso do Sul, Corumbá | GQ366253 | Faivovich et al. 2010 |
| *Phyllomedusa burmeisteri* | CFBH 17360 | Brazil, Minas Gerais, Furnas | GQ366256 | Faivovich et al. 2010 |
| *Phyllomedusa camba* | CFBH 17278 | Brazil, Rondônia, Ministro Andreazza | GQ366259 | Faivovich et al. 2010 |
| *Pithecopus centralis* | CHUNB 12570 | Brazil, Mato Grosso, Chapada dos Guimarães | GQ366260 | Faivovich et al. 2010 |
| *Phyllomedusa distincta* | CFBH 2658 | Brazil, Paraná, Guaratuba | GQ366262 | Faivovich et al. 2010 |
| *Pithecopus hypochondrialis* | AMNH A-141109 | Guyana, Dubulay, Berbice River | AY843724 | Faivovich et al. 2005 |
| *Pithecopus hypochondrialis* | ZUEC 16196 | Brazil, Amapá, Laranjal do Jari | KC520726 | Bruschi et al. 2013 |
| *Pithecopus hypochondrialis* | SMRP 32204 | Brazil, Amapá, Laranjal do Jari | MF926309 | Present study |
| *Pithecopus hypochondrialis* | SMRP 32208 | Brazil, Amapá, Laranjal do Jari | MF926310 | Present study |
| *Pithecopus hypochondrialis* | SMRP 32209 | Brazil, Amapá, Laranjal do Jari | MF926311 | Present study |
| *Pithecopus hypochondrialis* | SMRP 32203 | Brazil, Amapá, Laranjal do Jari | MF926308 | Present study |
| *Pithecopus hypochondrialis* | ZUEC16512 | Brazil, Pará, Prainha | KC520722 | Bruschi et al. 2013 |
| *Pithecopus hypochondrialis* | ZUEC 16511 | Brazil, Pará, Prainha | KC520721 | Bruschi et al. 2013 |
| *Pithecopus hypochondrialis* | ZUEC 19914 | Brazil, Pará, Óbidos | KC520706 | Bruschi et al. 2013 |
| *Pithecopus hypochondrialis* | ZUEC 19921 | Brazil, Pará, Óbidos | KC520728 | Bruschi et al. 2013 |
| *Pithecopus hypochondrialis* | ZUEC 13492 | Brazil, Tocantins, Porto Nacional | KC520707 | Bruschi et al. 2013 |
| *Pithecopus hypochondrialis* | ZUEC 16226 | Brazil, Maranhão, Bacabeira | KC520718 | Bruschi et al. 2013 |
| *Pithecopus hypochondrialis* | ZUEC 16237 | Brazil, Maranhão, Bacabeira | KC520768 | Bruschi et al. 2013 |
| *Pithecopus hypochondrialis* | SMRP 36732 | Brazil, Pará, Alenquer | MF926302 | Present study |
| *Pithecopus hypochondrialis* | SMRP 36736 | Brazil, Pará, Alenquer | MF926303 | Present study |
| *Pithecopus hypochondrialis* | SMRP 36737 | Brazil, Pará, Óbidos | MF926304 | Present study |
| *Pithecopus hypochondrialis* | CFBHt 167 | Brazil, Goiás, Silvânia | MF926306 | Present study |
| *Pithecopus hypochondrialis* | CFBHt 169 | Brazil, Goiás, Silvânia | MF926307 | Present study |
| *Pithecopus hypochondrialis* | CFBHt 164 | Brazil, Goiás, Silvânia | MF926305 | Present study |
| *Pithecopus hypochondrialis* | CFBHt 8290 | Brazil, Maranhão, Alcântara | MF926314 | Present study |
| *Pithecopus hypochondrialis* | AAG-UFU 1083 | Brazil, Mato Grosso, Barra do Garças | MF926333 | Present study |
| *Pithecopus hypochondrialis* | AAG-UFU 1084 | Brazil, Mato Grosso, Barra do Garças | MF926332 | Present study |
| *Pithecopus hypochondrialis* | ZUEC 15889 | Brazil, Minas Gerais, Uberlândia | MF926301 | Present study |
| *Phyllomedusa iheringii* | MNRJ 18782 | Brazil, Rio Grande do Sul, Santa Maria | GQ366264 | Faivovich et al. 2010 |
| *Pithecopus megacephalus* | MCNAM 6339 | Brazil, Minas Gerais, Serra do Cipó | GQ366267 | Faivovich et al. 2010 |
| *Phyllomedusa neildi* | CVULA 6503 | Venezuela, Falcon, Petit | GQ366270 | Faivovich et al. 2010 |
| *Pithecopus nordestinus* | CFBH 7330 | Brazil, Alagoas, Passo de Camarajibe | GQ366271 | Faivovich et al. 2010 |
| *Pithecopus nordestinus* | ZUEC 17832 | Brazil, Bahia, Alagoinhas | MF926316 | Present study |
| *Pithecopus nordestinus* | ZUEC 17833 | Brazil, Bahia, Alagoinhas | MF926317 | Present study |
| *Pithecopus nordestinus* | ZUEC 18625 | Brazil, Bahia, Alagoinhas | MF926318 | Present study |
| *Pithecopus nordestinus* | ZUEC 18626 | Brazil, Bahia, Alagoinhas | MF926320 | Present study |
| *Pithecopus nordestinus* | ZUEC 19909 | Brazil, Sergipe, Areia Branca | MF926319 | Present study |
| *Pithecopus nordestinus* | ZUEC 19905 | Brazil, Sergipe, Laranjeiras | MF926321 | Present study |
| *Pithecopus nordestinus* | ZUEC 19895 | Brazil, Sergipe, Laranjeiras | MF926322 | Present study |
| *Pithecopus nordestinus* | CFBHt 5367 | Brazil, Ceará, Ubajara | MF926313 | Present study |
| *Pithecopus nordestinus* | CFBHt 9391 | Brazil, Bahia, Maracás | MF926315 | Present study |
| *Pithecopus oreades* | CHUNB 56875 | Brazil, Goiás, Caldas Novas | GQ366278 | Faivovich et al. 2010 |
| *Pithecopus palliatus* | To be deposited in SMRP | Bolívia, Beni, Rurrenabaque | GQ366280 | Faivovich et al. 2010 |
| *Pithecopus “rohdei”* | CFBHt 93 | Brazil, São Paulo, Ubatuba | GQ366237 | Faivovich et al. 2010 |
| *Pithecopus “rohdei”* | CRR-18 | Brazil, Minas Gerais, Perdizes | GQ366240 | Faivovich et al. 2010 |
| *Pithecopus rusticus* | UFMG 13355 | Brazil, Santa Catarina, Água Doce | KM206705 | Bruschi et al. 2014 |
| *Pithecopus rusticus* | UFMG 13356 | Brazil, Santa Catarina, Água Doce | KM206706 | Bruschi et al. 2014 |
| *Pithecopus rusticus* | UFMG 13357 | Brazil, Santa Catarina, Água Doce | KM206707 | Bruschi et al. 2014 |
| *Pithecopus rusticus* | UFMG 13360 | Brazil, Santa Catarina, Água Doce | KM206708 | Bruschi et al. 2014 |
| *Pithecopus rusticus* | UFMG 13354 | Brazil, Santa Catarina, Água Doce | KM520705 | Bruschi et al. 2014 |
| *Pithecopus rusticus* | UFMG 13353 | Brazil, Santa Catarina, Água Doce | KM520734 | Bruschi et al. 2014 |
| *Phyllomedusa sauvagii* | CFBH 2573 | Brazil, Mato Grosso do Sul, Corumbá | GQ366281 | Faivovich et al. 2010 |
| *Phyllomedusa tarsius* | MJH 67 | Brazil, Amazonas, Reserva Ducke | AY843726 | Faivovich et al. 2005 |
| *Phyllomedusa tetraploidea* | CFBH 1725 | Brazil, São Paulo, Ribeirão Branco | GQ366285 | Faivovich et al. 2010 |
| *Phyllomedusa trinitatis* | CVULA7086 | Venezuela, Miranda | GQ366287 | Faivovich et al. 2010 |
| *Phyllomedusa vaillantii* | AMNH A- 166288 | Guyana, Berbice River | AY549363 | Faivovich et al. 2005 |
| *Pithecopus araguaius* sp. n. | ZUEC 21659 | Brazil, Mato Grosso, Pontal do Araguaia | MF926298 | Present study |
| *Pithecopus araguaius* sp. n. | ZUEC 21660 | Brazil, Mato Grosso, Pontal do Araguaia | MF926299 | Present study |
| *Pithecopus araguaius* sp. n. | ZUEC 21657 | Brazil, Mato Grosso, Pontal do Araguaia | MF926300 | Present study |
| *Pithecopus araguaius* sp. n. | ZUEC 13503 | Brazil, Mato Grosso, Santa Terezinha | KC520708 | Bruschi et al. 2013 |
| *Pithecopus araguaius* sp. n. | CFBHt 4638 | Brazil, Mato Grosso, Chapada dos Guimarães | MF926329 | Present study |
| *Pithecopus araguaius* sp. n. | CFBHt 4687 | Brazil, Mato Grosso, Chapada dos Guimarães | MF926331 | Present study |
| *Pithecopus araguaius* sp. n. | CFBHt 4684 | Brazil, Mato Grosso, Chapada dos Guimarães | MF926330 | Present study |

**Abreviation to collections.** Abreviation to collections: AMNH (American Museum of Natural History, USA); AAG-UFU (Collection of frogs of the Museu de Biodiversidade do Cerrado, Universidade Federal de Uberlãndia, Brazil); CFBH (Célio F. B. Haddad, Universidade Estadual Paulista, Brazil); CFBHt (Coleção de tecidos “Célio F. B. Haddad”, Universidade Estadual Paulista, Brazil); CHUNB (Coleção Herpetológica da Universidade Nacional de Brasília, Brazil); CRR (Camila R. Rabelo Field Series); CVULA (Vertebrados Universidad de los Andes, Venezuela); MACN (Museo Argentino de Ciencias Naturales Bernardino Rivadavia, Argentina); MCNAM (Museu de Ciências Naturais, PUC- MG, Brazil); MNRJ (Museu Nacional do Rio de Janeiro, Universidade Federal do Rio de Janeiro, Brazil); UFMG (Coleção Herpetológica da Universidade Federal de Minas Gerais, Brazil); ZUEC (Museu de Zoologia da Unicamp, Universidade Estadual de Campinas, Brazil); SMRP (Shirlei Maria Recco-Pimentel, Universidade Estadual de Campinas, Brazil); ZUFRJ (Museu Zoologia da Universidade Federal do Rio de Janeiro, Brazil).
