# Supplementary material for: Unrevealing the leaf frogs Cerrado diversity: A new species of Pithecopus (Anura, Arboranae, Phyllomedusidae) from the Mato Grosso state, Brazil
Source: PLoS One. 2017 Sep 27;12(9):e0184631. doi: 10.1371/journal.pone.0184631 (PMC5617161; doi:10.1371/journal.pone.0184631)
Supplement: S4 Table — Abbreviation to collections: ZUEC (Museu de Zoologia da Unicamp, Universidade Estadual de Campinas, Brazil); AAG-UFU (Collection of frogs of the Museu de Biodiversidade do Cerrado, Universidade Federal de Uberlândia, Brazil); MNRJ (Museu Nacional do Rio de Janeiro, Universidade Federal do Rio de Janeiro, Brazil); CFBH (Célio F. B. Haddad, Universidade Estadual Paulista, Brazil). Morphometric traits: snout-vent length (SVL), hand length (HAL), forearm length (FAL), thigh length (THL), foot length (FL), head length (HL), head width (HW), eye diameter (ED), internarial distance (IND), tibia length (TL) (= shank length), tympanum diameter (TD), and eye-nostril distance (END), upper arm length (UAL), tarsus length (TAL), the disc diameters of third finger (3FD), fourth finger (4FD), fourth toe (4TD), fifth toe (5TD), and axilla-groin length (AGL). (DOC) [file pone.0184631.s006.doc]

S4 Table

Raw morphological measurement data (values in millimeters) of examined *Pithecopus* specimens which were used for statistical analysis.

| Specimen | Species | SVL | HL | HW | AGL | ED | TD | END | IND | UAL | FAL | HAL | THL | TL | TAL | FL | 3FD | 4FD | 4TD | 5TD | Type specimen |
| --- | --- | --- | --- | --- | --- | --- | --- | --- | --- | --- | --- | --- | --- | --- | --- | --- | --- | --- | --- | --- | --- |
| ZUEC 16605 | *P. hypochondrialis* (North) | 39.9 | 8.6 | 12.1 | 19.3 | 5.1 | 2.0 | 2.6 | 3.4 | 7.3 | 9.1 | 10.5 | 16.5 | 17.6 | 13.0 | 13.6 | 1.3 | 1.2 | 1.4 | 1.3 | - |
| ZUEC 16504 | *P. hypochondrialis* (North) | 42.0 | 9.4 | 13.0 | 22.2 | 5.3 | 1.4 | 3.0 | 3.9 | 8.4 | 9.6 | 10.7 | 18.5 | 17.8 | 13.3 | 14.0 | 1.1 | 1.4 | 1.2 | 1.4 | - |
| ZUEC 16505 | *P. hypochondrialis* (North) | 39.6 | 8.0 | 12.5 | 20.3 | 5.0 | 1.8 | 2.6 | 3.5 | 8.4 | 9.8 | 10.2 | 18.7 | 17.5 | 13.7 | 13.8 | 1.6 | 1.6 | 1.6 | 1.6 | - |
| ZUEC 16506 | *P. hypochondrialis* (North) | 40.4 | 8.4 | 12.4 | 19.3 | 5.1 | 1.8 | 3.0 | 3.4 | 8.1 | 9.7 | 10.2 | 18.5 | 19.3 | 12.9 | 12.7 | 1.5 | 1.5 | 1.4 | 1.4 | - |
| ZUEC 16507 | *P. hypochondrialis* (North) | 38.9 | 9.1 | 12.4 | 18.7 | 4.7 | 1.7 | 2.8 | 3.7 | 8.0 | 9.5 | 9.7 | 17.0 | 18.5 | 12.3 | 14.1 | 1.5 | 1.4 | 1.4 | 1.5 | - |
| ZUEC 16508 | *P. hypochondrialis* (North) | 39.5 | 8.3 | 12.8 | 20.0 | 4.8 | 1.8 | 2.7 | 3.6 | 7.6 | 9.4 | 10.3 | 17.5 | 19.1 | 12.3 | 13.3 | 1.4 | 1.4 | 1.3 | 1.5 | - |
| ZUEC 16509 | *P. hypochondrialis* (North) | 39.9 | 8.5 | 13.0 | 21.5 | 4.9 | 2.3 | 3.0 | 3.6 | 8.3 | 9.4 | 10.3 | 18.1 | 19.3 | 13.6 | 13.3 | 1.6 | 1.6 | 1.5 | 1.7 | - |
| ZUEC 16550 | *P. hypochondrialis* (North) | 37.9 | 8.1 | 11.4 | 18.4 | 4.5 | 2.3 | 2.4 | 3.3 | 8.3 | 9.1 | 9.5 | 17.2 | 17.2 | 11.6 | 12.8 | 1.4 | 1.2 | 1.4 | 1.6 | - |
| ZUEC 16555 | *P. hypochondrialis* (North) | 36.6 | 7.7 | 11.2 | 16.9 | 4.7 | 2.1 | 2.7 | 3.2 | 7.4 | 8.0 | 9.8 | 16.8 | 16.1 | 10.3 | 12.1 | 1.4 | 1.3 | 1.4 | 1.6 | - |
| ZUEC 16562 | *P. hypochondrialis* (North) | 37.3 | 7.7 | 11.4 | 18.6 | 4.5 | 1.7 | 2.7 | 3.4 | 8.2 | 8.7 | 10.2 | 16.8 | 16.3 | 11.0 | 12.5 | 1.6 | 1.6 | 1.5 | 1.7 | - |
| ZUEC 16559 | *P. hypochondrialis* (North) | 37.6 | 8.0 | 11.4 | 18.8 | 4.4 | 1.7 | 2.5 | 3.3 | 7.7 | 8.3 | 10.1 | 17.4 | 17.3 | 12.5 | 12.7 | 1.0 | 1.1 | 1.1 | 1.2 | - |
| ZUEC 16553 | *P. hypochondrialis* (North) | 38.5 | 9.0 | 11.9 | 19.1 | 4.5 | 1.8 | 2.7 | 3.6 | 7.6 | 9.1 | 10.2 | 16.4 | 17.2 | 10.3 | 12.7 | 1.3 | 1.3 | 1.3 | 1.4 | - |
| ZUEC 16551 | *P. hypochondrialis* (North) | 39.7 | 8.4 | 12.0 | 20.2 | 4.8 | 1.4 | 2.8 | 3.4 | 7.8 | 9.8 | 10.1 | 17.4 | 17.5 | 12.0 | 13.3 | 1.3 | 1.5 | 1.3 | 1.3 | - |
| ZUEC 16511 | *P. hypochondrialis* (North) | 36.4 | 8.1 | 10.8 | 19.3 | 4.3 | 1.9 | 2.5 | 3.2 | 7.3 | 8.1 | 9.1 | 15.5 | 16.2 | 11.0 | 12.2 | 1.0 | 1.1 | 1.0 | 1.3 | - |
| ZUEC 16530 | *P. hypochondrialis* (North) | 35.4 | 8.0 | 11.3 | 16.1 | 4.0 | 1.4 | 2.5 | 3.3 | 8.0 | 9.0 | 9.4 | 17.0 | 17.0 | 11.4 | 11.6 | 1.0 | 0.9 | 0.9 | 0.9 | - |
| ZUEC 16529 | *P. hypochondrialis* (North) | 33.6 | 7.6 | 10.5 | 16.3 | 4.3 | 1.6 | 2.3 | 3.5 | 6.8 | 7.6 | 8.9 | 14.9 | 14.8 | 9.9 | 11.4 | 1.0 | 1.0 | 1.0 | 1.0 | - |
| ZUEC 16561 | *P. hypochondrialis* (North) | 36.5 | 8.0 | 11.6 | 16.6 | 4.3 | 1.7 | 2.3 | 3.3 | 7.4 | 8.5 | 8.4 | 16.9 | 16.1 | 11.7 | 12.1 | 1.6 | 1.4 | 1.4 | 1.5 | - |
| ZUEC 16520 | *P. hypochondrialis* (North) | 36.5 | 8.7 | 11.4 | 16.6 | 4.1 | 1.8 | 2.5 | 3.4 | 7.1 | 8.9 | 9.7 | 17.3 | 16.8 | 11.8 | 12.3 | 1.2 | 1.3 | 1.2 | 1.3 | - |
| ZUEC 16515 | *P. hypochondrialis* (North) | 36.7 | 8.4 | 11.5 | 17.5 | 4.4 | 1.8 | 2.6 | 3.4 | 7.4 | 8.3 | 9.5 | 15.2 | 16.1 | 10.8 | 12.6 | 1.2 | 1.1 | 1.3 | 1.4 | - |
| ZUEC 19916 | *P. hypochondrialis* (North) | 37.6 | 8.6 | 11.6 | 19.3 | 4.5 | 1.8 | 2.4 | 3.7 | 7.7 | 8.5 | 9.7 | 16.2 | 16.1 | 11.4 | 10.7 | 1.3 | 1.1 | 1.2 | 1.3 | - |
| ZUEC 19940 | *P. hypochondrialis* (North) | 38.0 | 9.3 | 12.4 | 18.9 | 4.8 | 1.6 | 2.5 | 3.4 | 7.4 | 8.6 | 9.1 | 16.5 | 15.9 | 11.4 | 11.8 | 1.0 | 0.9 | 0.9 | 1.5 | - |
| ZUEC 19944 | *P. hypochondrialis* (North) | 33.7 | 7.1 | 11.0 | 15.0 | 4.1 | 1.5 | 2.1 | 3.1 | 7.2 | 7.8 | 9.0 | 15.1 | 15.7 | 10.9 | 12.1 | 1.3 | 1.3 | 1.4 | 1.4 | - |
| ZUEC 19938 | *P. hypochondrialis* (North) | 36.9 | 7.2 | 11.1 | 18.1 | 4.2 | 1.4 | 2.6 | 3.3 | 7.4 | 7.5 | 8.3 | 16.2 | 15.8 | 11.4 | 11.5 | 1.3 | 1.0 | 1.1 | 1.0 | - |
| ZUEC 19917 | *P. hypochondrialis* (North) | 34.0 | 7.3 | 10.6 | 15.7 | 3.7 | 2.0 | 2.3 | 3.1 | 7.0 | 7.8 | 8.9 | 15.3 | 15.6 | 10.3 | 12.3 | 1.1 | 1.1 | 1.1 | 1.2 | - |
| ZUEC 19930 | *P. hypochondrialis* (North) | 34.6 | 7.8 | 10.8 | 16.8 | 4.1 | 2.0 | 2.5 | 3.3 | 7.7 | 8.3 | 9.1 | 15.6 | 15.4 | 10.5 | 11.7 | 1.1 | 1.2 | 1.0 | 1.1 | - |
| ZUEC 19932 | *P. hypochondrialis* (North) | 35.1 | 7.3 | 11.3 | 16.7 | 4.2 | 2.0 | 2.6 | 3.5 | 7.5 | 8.9 | 9.6 | 16.4 | 15.9 | 11.3 | 11.9 | 1.2 | 1.2 | 1.2 | 1.4 | - |
| ZUEC 19923 | *P. hypochondrialis* (North) | 35.8 | 7.4 | 11.2 | 16.3 | 4.3 | 2.0 | 2.5 | 3.6 | 7.2 | 7.8 | 9.7 | 15.8 | 15.2 | 10.3 | 11.6 | 1.2 | 1.1 | 1.4 | 1.3 | - |
| ZUEC 19927 | *P. hypochondrialis* (North) | 36.2 | 7.4 | 10.9 | 18.4 | 3.6 | 1.5 | 2.4 | 3.6 | 7.2 | 7.9 | 10.5 | 15.8 | 15.7 | 10.5 | 12.4 | 1.3 | 1.3 | 1.0 | 1.2 | - |
| ZUEC 19920 | *P. hypochondrialis* (North) | 34.3 | 7.2 | 10.5 | 17.0 | 4.3 | 1.8 | 2.2 | 3.3 | 7.7 | 7.9 | 9.3 | 15.7 | 15.4 | 10.5 | 11.8 | 1.1 | 1.2 | 1.2 | 1.1 | - |
| ZUEC 19922 | *P. hypochondrialis* (North) | 37.3 | 8.4 | 12.1 | 17.5 | 4.7 | 2.0 | 2.4 | 3.7 | 7.2 | 8.4 | 9.6 | 17.6 | 16.9 | 11.4 | 11.7 | 1.3 | 1.2 | 1.3 | 1.4 | - |
| ZUEC 19926 | *P. hypochondrialis* (North) | 36.2 | 7.9 | 11.4 | 17.2 | 4.5 | 2.0 | 2.6 | 3.4 | 6.6 | 8.2 | 9.3 | 16.0 | 15.4 | 10.8 | 11.7 | 1.4 | 1.3 | 1.3 | 1.5 | - |
| ZUEC 19928 | *P. hypochondrialis* (North) | 37.4 | 8.0 | 11.5 | 17.5 | 4.7 | 1.9 | 2.5 | 3.5 | 7.4 | 9.3 | 10.2 | 17.5 | 16.6 | 11.0 | 13.6 | 1.5 | 1.4 | 1.5 | 1.5 | - |
| ZUEC 19918 | *P. hypochondrialis* (North) | 36.8 | 7.6 | 11.3 | 17.5 | 4.2 | 1.5 | 2.5 | 3.5 | 7.1 | 8.8 | 10.2 | 16.9 | 16.9 | 11.3 | 13.3 | 1.3 | 1.3 | 1.3 | 1.2 | - |
| ZUEC 19921 | *P. hypochondrialis* (North) | 35.1 | 9.2 | 11.4 | 16.0 | 4.5 | 1.7 | 2.5 | 3.7 | 7.4 | 8.9 | 10.1 | 17.1 | 16.5 | 11.6 | 11.2 | 1.3 | 1.3 | 1.4 | 1.5 | - |
| ZUEC 19945 | *P. hypochondrialis* (North) | 36.5 | 8.9 | 12.4 | 20.3 | 4.1 | 2.1 | 2.7 | 3.3 | 8.0 | 8.7 | 10.7 | 16.0 | 16.2 | 11.7 | 12.8 | 1.2 | 1.0 | 1.0 | 1.2 | - |
| ZUEC 19943 | *P. hypochondrialis* (North) | 37.7 | 8.6 | 11.4 | 18.2 | 4.3 | 1.7 | 2.8 | 3.6 | 7.5 | 7.6 | 9.5 | 16.1 | 15.7 | 10.7 | 12.8 | 0.8 | 0.8 | 0.9 | 0.9 | - |
| ZUEC 19933 | *P. hypochondrialis* (North) | 36.3 | 9.2 | 11.8 | 15.8 | 4.4 | 2.3 | 2.4 | 3.4 | 7.7 | 9.4 | 9.9 | 17.5 | 16.4 | 12.1 | 11.8 | 1.2 | 1.2 | 1.1 | 1.2 | - |
| AAG-UFU 5987 | *P. hypochondrialis* (North) | 36.6 | 9.1 | 11.5 | 16.4 | 4.8 | 1.5 | 2.5 | 3.4 | 7.7 | 8.4 | 9.9 | 16.8 | 16.3 | 12.0 | 12.5 | 1.1 | 1.1 | 1.1 | 1.2 | - |
| AAG-UFU 5988 | *P. hypochondrialis* (North) | 36.1 | 9.3 | 11.3 | 14.8 | 4.7 | 1.8 | 2.8 | 3.7 | 8.1 | 8.9 | 9.6 | 17.5 | 17.3 | 11.5 | 12.3 | 1.3 | 1.3 | 1.4 | 1.5 | - |
| AAG-UFU 5989 | *P. hypochondrialis* (North) | 38.1 | 8.8 | 11.6 | 14.7 | 4.5 | 2.2 | 2.5 | 3.5 | 7.3 | 8.8 | 9.6 | 17.4 | 17.1 | 11.2 | 12.6 | 1.3 | 1.4 | 1.4 | 1.5 | - |
| AAG-UFU 5998 | *P. hypochondrialis* (North) | 38.0 | 8.6 | 11.7 | 18.6 | 4.4 | 2.3 | 2.6 | 3.5 | 7.5 | 8.8 | 10.3 | 17.2 | 16.9 | 11.5 | 12.9 | 1.5 | 1.5 | 1.6 | 1.7 | - |
| AAG-UFU 5999 | *P. hypochondrialis* (North) | 38.0 | 9.7 | 11.7 | 17.5 | 4.8 | 2.1 | 2.7 | 3.7 | 7.7 | 9.3 | 10.1 | 16.8 | 16.3 | 10.9 | 13.0 | 1.4 | 1.5 | 1.5 | 1.6 | - |
| AAG-UFU 6000 | *P. hypochondrialis* (North) | 39.0 | 9.2 | 12.0 | 18.3 | 4.5 | 1.9 | 2.7 | 3.6 | 7.8 | 8.9 | 10.5 | 16.7 | 16.9 | 11.8 | 12.8 | 1.5 | 1.5 | 1.6 | 1.7 | - |
| AAG-UFU 3489 | *P. hypochondrialis* (South) | 34.2 | 7.6 | 10.8 | 15.5 | 4.0 | 1.7 | 2.3 | 3.3 | 7.3 | 8.0 | 9.3 | 14.4 | 15.3 | 10.4 | 11.6 | 1.1 | 1.1 | 1.1 | 1.3 | - |
| AAG-UFU 3490 | *P. hypochondrialis* (South) | 36.5 | 8.9 | 10.5 | 16.3 | 4.9 | 2.0 | 2.3 | 3.5 | 7.7 | 8.3 | 9.0 | 15.5 | 15.5 | 10.4 | 12.7 | 1.0 | 1.1 | 1.2 | 1.3 | - |
| AAG-UFU 3491 | *P. hypochondrialis* (South) | 34.6 | 7.8 | 10.7 | 15.5 | 3.9 | 2.0 | 2.6 | 2.8 | 7.6 | 8.6 | 9.4 | 15.8 | 15.2 | 10.4 | 12.8 | 0.9 | 0.9 | 1.0 | 1.1 | - |
| AAG-UFU 3492 | *P. hypochondrialis* (South) | 35.8 | 7.4 | 11.2 | 19.4 | 4.1 | 1.8 | 2.7 | 3.1 | 8.1 | 8.3 | 9.4 | 15.2 | 14.9 | 10.5 | 12.5 | 1.2 | 1.1 | 1.1 | 1.1 | - |
| AAG-UFU 3493 | *P. hypochondrialis* (South) | 36.2 | 8.1 | 11.2 | 17.2 | 4.3 | 1.9 | 2.7 | 3.4 | 7.4 | 8.6 | 9.6 | 15.9 | 15.8 | 10.4 | 13.3 | 1.2 | 1.1 | 1.1 | 1.3 | - |
| AAG-UFU 3494 | *P. hypochondrialis* (South) | 37.7 | 7.9 | 11.7 | 18.8 | 4.3 | 1.9 | 2.8 | 3.4 | 7.9 | 8.2 | 9.8 | 15.4 | 15.7 | 10.3 | 12.2 | 1.2 | 1.2 | 1.3 | 1.3 | - |
| AAG-UFU 21650 | *P. hypochondrialis* (South) | 36.6 | 7.8 | 11.7 | 18.3 | 4.2 | 1.9 | 2.2 | 3.3 | 6.9 | 7.8 | 9.2 | 15.7 | 15.2 | 10.6 | 11.9 | 1.0 | 0.9 | 0.8 | 1.0 | - |
| AAG-UFU 1078 | *P. hypochondrialis* (South) | 36.3 | 8.0 | 10.9 | 14.9 | 4.0 | 2.0 | 2.3 | 3.8 | 7.3 | 7.9 | 9.3 | 15.1 | 15.3 | 10.7 | 12.7 | 1.0 | 1.2 | 1.1 | 1.4 | - |
| AAG-UFU 1079 | *P. hypochondrialis* (South) | 34.6 | 8.4 | 10.8 | 14.5 | 4.0 | 1.5 | 2.2 | 3.2 | 7.2 | 6.9 | 9.4 | 14.9 | 15.9 | 11.2 | 12.4 | 1.0 | 1.0 | 1.1 | 1.1 | - |
| AAG-UFU 1080 | *P. hypochondrialis* (South) | 36.5 | 8.8 | 11.0 | 15.8 | 4.6 | 1.6 | 2.5 | 3.8 | 7.9 | 9.0 | 9.7 | 15.9 | 16.5 | 11.2 | 12.4 | 0.8 | 1.1 | 1.2 | 1.4 | - |
| AAG-UFU 1081 | *P. hypochondrialis* (South) | 35.0 | 9.2 | 11.0 | 15.0 | 4.5 | 1.5 | 2.2 | 3.5 | 7.5 | 7.6 | 9.4 | 15.2 | 15.6 | 10.7 | 12.4 | 1.1 | 1.2 | 1.2 | 1.2 | - |
| AAG-UFU 1082 | *P. hypochondrialis* (South) | 35.1 | 7.3 | 10.7 | 15.0 | 4.3 | 2.0 | 2.2 | 3.4 | 7.5 | 8.4 | 9.8 | 15.7 | 15.8 | 11.0 | 12.2 | 0.9 | 1.0 | 1.1 | 1.2 | - |
| AAG-UFU 1083 | *P. hypochondrialis* (South) | 36.8 | 8.1 | 11.4 | 17.2 | 3.8 | 1.6 | 2.3 | 3.6 | 8.3 | 8.8 | 9.6 | 16.3 | 16.3 | 11.0 | 11.9 | 1.1 | 1.1 | 1.1 | 1.2 | - |
| AAG-UFU 1084 | *P. hypochondrialis* (South) | 37.4 | 8.7 | 11.0 | 16.7 | 4.5 | 1.9 | 2.3 | 3.6 | 7.9 | 9.0 | 9.6 | 16.1 | 16.3 | 11.3 | 13.3 | 1.1 | 1.1 | 1.1 | 1.2 | - |
| AAG-UFU 1963 | *P. hypochondrialis* (South) | 33.7 | 5.9 | 10.2 | 16.3 | 3.9 | 1.7 | 2.4 | 2.9 | 6.6 | 7.8 | 10.0 | 13.2 | 13.8 | 9.8 | 12.1 | 0.9 | 0.9 | 1.0 | 1.1 | - |
| AAG-UFU 1964 | *P. hypochondrialis* (South) | 36.8 | 7.0 | 11.7 | 16.0 | 4.7 | 1.2 | 2.2 | 3.8 | 8.1 | 8.0 | 10.3 | 14.9 | 15.8 | 11.0 | 12.2 | 1.0 | 1.0 | 1.1 | 1.2 | - |
| AAG-UFU 991 | *P. hypochondrialis* (South) | 33.5 | 6.2 | 10.1 | 14.3 | 4.2 | 1.5 | 2.2 | 3.0 | 7.5 | 7.6 | 9.4 | 14.5 | 14.7 | 9.5 | 12.2 | 1.0 | 1.0 | 1.0 | 1.2 | - |
| AAG-UFU 992 | *P. hypochondrialis* (South) | 35.1 | 7.3 | 10.4 | 16.6 | 4.3 | 1.6 | 2.1 | 2.8 | 7.4 | 7.8 | 9.2 | 14.8 | 15.0 | 10.0 | 12.4 | 0.9 | 1.0 | 1.2 | 1.2 | - |
| AAG-UFU 993 | *P. hypochondrialis* (South) | 34.9 | 7.2 | 10.8 | 16.2 | 5.0 | 1.9 | 2.4 | 3.2 | 7.2 | 8.8 | 9.3 | 14.9 | 15.5 | 10.1 | 12.5 | 0.9 | 1.0 | 1.0 | 1.1 | - |
| AAG-UFU 996 | *P. hypochondrialis* (South) | 34.1 | 6.4 | 10.6 | 14.5 | 4.2 | 1.8 | 2.5 | 2.9 | 7.2 | 7.7 | 8.8 | 15.6 | 15.0 | 10.5 | 12.0 | 0.7 | 1.0 | 1.0 | 1.2 | - |
| AAG-UFU 997 | *P. hypochondrialis* (South) | 34.8 | 6.8 | 10.4 | 15.9 | 4.1 | 1.7 | 2.2 | 2.9 | 7.0 | 7.7 | 9.2 | 14.8 | 14.3 | 9.6 | 12.6 | 1.1 | 1.0 | 1.2 | 1.3 | - |
| AAG-UFU 998 | *P. hypochondrialis* (South) | 35.0 | 6.4 | 10.4 | 16.8 | 4.5 | 1.7 | 2.2 | 3.4 | 7.0 | 8.4 | 9.5 | 15.5 | 15.7 | 10.6 | 13.0 | 1.1 | 1.0 | 1.1 | 1.1 | - |
| AAG-UFU 999 | *P. hypochondrialis* (South) | 33.3 | 6.2 | 10.8 | 15.2 | 4.5 | 2.0 | 2.1 | 3.2 | 7.6 | 8.2 | 9.1 | 15.2 | 14.8 | 10.2 | 12.7 | 0.9 | 0.9 | 0.9 | 1.0 | - |
| AAG-UFU 331 | *P. hypochondrialis* (South) | 34.9 | 7.2 | 10.9 | 14.1 | 4.5 | 1.9 | 2.3 | 3.6 | 7.2 | 8.2 | 8.7 | 14.7 | 14.6 | 10.7 | 11.8 | 0.9 | 1.2 | 1.1 | 1.3 | - |
| AAG-UFU 334 | *P. hypochondrialis* (South) | 33.4 | 7.3 | 10.8 | 14.6 | 4.3 | 1.5 | 2.3 | 3.5 | 7.2 | 7.8 | 8.6 | 13.9 | 14.3 | 10.8 | 12.0 | 1.1 | 1.1 | 1.1 | 1.2 | - |
| AAG-UFU 0117 | *P. hypochondrialis* (South) | 32.5 | 7.4 | 10.2 | 14.3 | 4.4 | 1.5 | 2.1 | 3.0 | 6.7 | 7.6 | 8.4 | 14.5 | 14.0 | 9.9 | 11.7 | 1.0 | 1.1 | 1.0 | 1.1 | - |
| AAG-UFU 0118 | *P. hypochondrialis* (South) | 34.0 | 7.8 | 11.3 | 15.4 | 4.5 | 1.9 | 2.3 | 3.2 | 7.7 | 8.1 | 9.2 | 14.7 | 14.4 | 10.6 | 12.2 | 1.0 | 1.1 | 1.1 | 1.5 | - |
| AAG-UFU 1333 | *P. hypochondrialis* (South) | 35.5 | 8.0 | 11.1 | 16.3 | 4.7 | 1.9 | 2.3 | 3.4 | 6.7 | 8.4 | 9.2 | 14.6 | 14.5 | 10.8 | 12.0 | 1.0 | 1.0 | 0.9 | 1.1 | - |
| AAG-UFU 3410 | *P. hypochondrialis* (South) | 36.6 | 8.8 | 10.7 | 18.2 | 4.2 | 1.8 | 2.4 | 3.3 | 7.0 | 7.9 | 9.1 | 14.1 | 14.3 | 10.2 | 12.2 | 1.2 | 1.2 | 1.3 | 1.4 | - |
| AAG-UFU 2827 | *P. hypochondrialis* (South) | 38.4 | 9.2 | 11.7 | 20.1 | 4.1 | 2.2 | 2.4 | 3.8 | 7.3 | 9.1 | 9.7 | 15.6 | 15.8 | 11.4 | 12.8 | 1.1 | 1.1 | 1.1 | 1.2 | - |
| AAG-UFU 2828 | *P. hypochondrialis* (South) | 35.7 | 8.5 | 10.7 | 17.0 | 4.0 | 1.8 | 2.4 | 3.6 | 6.7 | 8.4 | 9.7 | 14.5 | 15.1 | 11.5 | 11.9 | 0.9 | 0.9 | 1.0 | 1.0 | - |
| AAG-UFU 2829 | *P. hypochondrialis* (South) | 39.5 | 9.4 | 12.1 | 17.7 | 4.6 | 2.3 | 2.8 | 3.9 | 7.7 | 8.1 | 10.1 | 15.2 | 15.7 | 11.0 | 12.6 | 1.2 | 1.0 | 1.2 | 1.2 | - |
| AAG-UFU 2830 | *P. hypochondrialis* (South) | 38.2 | 9.9 | 12.3 | 19.5 | 4.5 | 2.1 | 2.9 | 3.5 | 7.6 | 8.8 | 9.9 | 14.3 | 15.6 | 10.8 | 12.4 | 1.0 | 1.0 | 1.2 | 1.2 | - |
| AAG-UFU 2779 | *P. hypochondrialis* (South) | 34.7 | 8.5 | 10.5 | 16.1 | 4.1 | 2.0 | 2.4 | 2.9 | 7.6 | 7.6 | 9.4 | 14.2 | 14.1 | 9.8 | 11.5 | 1.0 | 0.9 | 1.1 | 1.1 | - |
| AAG-UFU 2817 | *P. hypochondrialis* (South) | 36.5 | 8.6 | 11.1 | 18.6 | 4.7 | 1.9 | 2.5 | 3.7 | 7.2 | 8.8 | 9.8 | 15.2 | 15.4 | 10.8 | 12.3 | 1.1 | 1.1 | 1.1 | 1.3 | - |
| AAG-UFU 2311 | *P. hypochondrialis* (South) | 35.5 | 7.8 | 10.7 | 16.1 | 4.8 | 1.7 | 2.5 | 3.1 | 7.9 | 8.5 | 9.2 | 13.9 | 14.7 | 10.7 | 12.6 | 1.0 | 1.2 | 1.2 | 1.2 | - |
| AAG-UFU 2313 | *P. hypochondrialis* (South) | 34.4 | 7.7 | 10.8 | 15.6 | 4.5 | 1.9 | 2.4 | 3.4 | 7.5 | 7.9 | 9.1 | 14.9 | 14.6 | 9.8 | 11.4 | 1.0 | 1.1 | 1.1 | 1.3 | - |
| AAG-UFU 2315 | *P. hypochondrialis* (South) | 35.7 | 7.9 | 11.1 | 17.4 | 4.8 | 1.8 | 2.6 | 3.1 | 6.9 | 8.2 | 9.3 | 14.6 | 15.2 | 10.5 | 11.9 | 1.2 | 1.4 | 1.3 | 1.5 | - |
| AAG-UFU 2299 | *P. hypochondrialis* (South) | 36.9 | 7.9 | 11.6 | 16.2 | 5.1 | 2.1 | 2.6 | 3.5 | 7.1 | 8.1 | 9.9 | 15.1 | 14.9 | 10.2 | 12.6 | 1.3 | 1.3 | 1.4 | 1.6 | - |
| AAG-UFU 3256 | *P. hypochondrialis* (South) | 33.7 | 7.6 | 11.2 | 17.1 | 4.4 | 1.4 | 2.4 | 3.4 | 7.8 | 7.2 | 8.5 | 13.7 | 13.6 | 10.4 | 11.4 | 1.0 | 1.1 | 1.1 | 1.1 | - |
| AAG-UFU 1276 | *P. hypochondrialis* (South) | 36.1 | 8.7 | 11.8 | 15.6 | 4.6 | 1.8 | 2.6 | 3.0 | 7.2 | 8.5 | 8.8 | 14.9 | 15.1 | 10.8 | 11.9 | 1.0 | 1.1 | 1.0 | 1.1 | - |
| AAG-UFU 4936 | *P. hypochondrialis* (South) | 36.2 | 8.3 | 11.8 | 14.9 | 4.6 | 1.2 | 2.5 | 3.4 | 7.3 | 8.2 | 9.4 | 15.8 | 15.5 | 11.4 | 12.9 | 1.1 | 1.0 | 1.2 | 1.2 | - |
| AAG-UFU 4937 | *P. hypochondrialis* (South) | 35.9 | 8.9 | 11.7 | 15.1 | 4.7 | 1.1 | 2.0 | 3.5 | 7.4 | 7.8 | 9.3 | 15.0 | 15.0 | 9.6 | 12.0 | 1.1 | 1.2 | 1.3 | 1.5 | - |
| AAG-UFU 4832 | *P. hypochondrialis* (South) | 36.1 | 8.4 | 11.7 | 16.3 | 4.6 | 1.7 | 2.4 | 3.5 | 7.1 | 8.8 | 10.2 | 15.5 | 15.3 | 10.6 | 12.5 | 1.2 | 1.3 | 1.1 | 1.3 | - |
| AAG-UFU 4834 | *P. hypochondrialis* (South) | 34.2 | 7.4 | 11.5 | 13.8 | 4.4 | 1.6 | 2.1 | 3.4 | 7.0 | 8.2 | 9.3 | 14.3 | 14.3 | 9.8 | 12.2 | 1.0 | 1.1 | 1.0 | 1.2 | - |
| AAG-UFU 4573 | *P. hypochondrialis* (South) | 35.6 | 7.8 | 10.6 | 16.2 | 4.4 | 1.4 | 2.4 | 3.3 | 6.8 | 8.0 | 8.8 | 14.2 | 14.5 | 11.1 | 12.2 | 1.0 | 1.0 | 1.1 | 1.2 | - |
| AAG-UFU 4689 | *P. hypochondrialis* (South) | 34.3 | 7.2 | 10.7 | 14.9 | 4.5 | 1.4 | 2.3 | 3.3 | 7.0 | 7.0 | 8.8 | 15.0 | 14.8 | 10.7 | 11.5 | 1.0 | 1.1 | 1.1 | 1.2 | - |
| AAG-UFU 3116 | *P. hypochondrialis* (South) | 33.9 | 7.0 | 10.2 | 15.0 | 4.3 | 1.5 | 1.9 | 3.3 | 7.3 | 6.7 | 8.6 | 14.7 | 14.3 | 10.3 | 11.8 | 0.9 | 1.0 | 1.0 | 1.2 | - |
| AAG-UFU 3101 | *P. hypochondrialis* (South) | 32.2 | 7.8 | 9.8 | 15.5 | 4.3 | 1.7 | 2.0 | 2.9 | 7.2 | 7.2 | 8.6 | 13.7 | 13.9 | 9.4 | 11.4 | 0.7 | 0.9 | 0.9 | 0.9 | - |
| AAG-UFU 455 | *P. hypochondrialis* (South) | 33.7 | 7.6 | 10.6 | 13.5 | 4.6 | 1.5 | 2.3 | 3.1 | 7.0 | 7.9 | 8.9 | 13.9 | 14.2 | 10.8 | 11.6 | 0.9 | 1.1 | 1.0 | 1.1 | - |
| AAG-UFU 3442 | *P. araguaius* sp. n. | 32.8 | 6.4 | 9.5 | 15.7 | 4.2 | 1.7 | 2.2 | 3.2 | 7.8 | 7.8 | 8.3 | 13.7 | 13.5 | 9.3 | 10.5 | 0.9 | 0.9 | 1.0 | 1.0 | paratopotype |
| AAG-UFU 3443 | *P. araguaius* sp. n. | 31.0 | 6.6 | 9.5 | 14.0 | 3.9 | 1.3 | 2.0 | 2.7 | 6.9 | 7.8 | 7.3 | 13.3 | 13.0 | 8.8 | 9.8 | 0.8 | 0.8 | 0.9 | 1.0 | paratopotype |
| AAG-UFU 3444 | *P. araguaius* sp. n. | 30.6 | 6.7 | 9.6 | 14.6 | 3.9 | 1.6 | 2.1 | 2.8 | 6.1 | 6.8 | 7.2 | 12.9 | 12.6 | 8.4 | 10.1 | 0.8 | 0.8 | 0.8 | 0.9 | holotype |
| AAG-UFU 3445 | *P. araguaius* sp. n. | 33.0 | 6.5 | 9.9 | 16.4 | 4.0 | 1.8 | 2.0 | 2.9 | 7.5 | 8.0 | 8.2 | 13.6 | 13.3 | 8.7 | 10.5 | 0.8 | 0.8 | 0.8 | 0.9 | paratopotype |
| AAG-UFU 3446 | *P. araguaius* sp. n. | 32.2 | 6.4 | 10.0 | 15.1 | 4.1 | 1.5 | 2.3 | 3.2 | 6.9 | 7.2 | 7.9 | 13.9 | 13.6 | 9.4 | 10.2 | 0.9 | 0.9 | 1.0 | 1.0 | paratopotype |
| AAG-UFU 3447 | *P. araguaius* sp. n. | 30.4 | 6.6 | 9.6 | 13.0 | 4.1 | 1.7 | 1.8 | 2.8 | 6.5 | 7.5 | 8.1 | 13.1 | 13.3 | 9.2 | 10.9 | 0.8 | 0.9 | 1.0 | 1.0 | paratopotype |
| AAG-UFU 3448 | *P. araguaius* sp. n. | 30.4 | 6.9 | 9.2 | 13.9 | 3.7 | 1.3 | 1.9 | 3.0 | 6.5 | 7.0 | 7.4 | 12.7 | 12.3 | 8.2 | 9.6 | 0.8 | 0.8 | 0.9 | 1.0 | paratopotype |
| AAG-UFU 3449 | *P. araguaius* sp. n. | 33.8 | 6.7 | 10.1 | 16.9 | 3.9 | 1.7 | 2.1 | 3.1 | 7.2 | 7.7 | 8.6 | 13.4 | 14.1 | 10.3 | 10.7 | 0.8 | 0.9 | 1.0 | 1.0 | paratopotype |
| AAG-UFU 4877 | *P. araguaius* sp. n. | 31.4 | 7.5 | 9.7 | 14.2 | 3.8 | 1.5 | 1.9 | 2.5 | 6.4 | 6.8 | 7.4 | 13.6 | 13.9 | 9.2 | 10.4 | 0.8 | 1.0 | 0.9 | 1.0 | paratopotype |
| AAG-UFU 4878 | *P. araguaius* sp. n. | 33.6 | 7.1 | 10.1 | 16.0 | 4.1 | 1.7 | 1.8 | 2.8 | 7.2 | 7.6 | 8.2 | 14.1 | 13.8 | 9.3 | 11.1 | 1.0 | 1.0 | 1.2 | 1.1 | paratopotype |
| AAG-UFU 4879 | *P. araguaius* sp. n. | 30.3 | 6.1 | 9.4 | 15.1 | 3.8 | 1.6 | 1.7 | 2.7 | 6.8 | 7.7 | 8.2 | 12.3 | 13.3 | 8.7 | 10.2 | 0.7 | 0.7 | 0.9 | 1.0 | paratopotype |
| AAG-UFU 4880 | *P. araguaius* sp. n. | 31.9 | 6.4 | 9.5 | 13.3 | 3.9 | 1.7 | 1.8 | 2.6 | 7.0 | 7.0 | 8.2 | 14.2 | 13.6 | 9.5 | 10.9 | 0.8 | 0.9 | 1.0 | 1.1 | paratopotype |
| AAG-UFU 4881 | *P. araguaius* sp. n. | 31.8 | 8.0 | 9.6 | 13.5 | 4.0 | 1.8 | 2.0 | 2.8 | 6.8 | 7.7 | 7.8 | 13.0 | 13.7 | 9.0 | 10.1 | 0.9 | 0.9 | 1.1 | 1.1 | paratopotype |
| AAG-UFU 4882 | *P. araguaius* sp. n. | 33.0 | 7.4 | 9.6 | 13.6 | 4.2 | 1.7 | 1.8 | 2.8 | 7.5 | 7.7 | 7.9 | 14.4 | 14.1 | 9.3 | 10.0 | 0.9 | 0.8 | 0.9 | 1.1 | paratopotype |
| ZUEC 21657 | *P. araguaius* sp. n. | 30.8 | 6.9 | 9.5 | 14.6 | 4.4 | 1.8 | 2.0 | 2.8 | 6.8 | 6.5 | 7.5 | 12.5 | 12.7 | 8.1 | 10.1 | 1.0 | 1.0 | 0.8 | 0.9 | paratopotype |
| ZUEC 21658 | *P. araguaius* sp. n. | 31.3 | 6.6 | 9.3 | 14.4 | 3.9 | 1.5 | 2.0 | 2.8 | 6.7 | 7.2 | 8.0 | 13.5 | 13.1 | 8.4 | 10.5 | 0.7 | 0.7 | 0.7 | 0.9 | paratopotype |
| ZUEC 21659 | *P. araguaius* sp. n. | 32.7 | 7.4 | 9.5 | 14.1 | 4.0 | 1.8 | 2.2 | 3.0 | 7.4 | 7.2 | 7.5 | 13.0 | 14.1 | 9.4 | 10.3 | 0.9 | 0.9 | 1.0 | 1.0 | paratopotype |
| ZUEC 21660 | *P. araguaius* sp. n. | 30.5 | 6.2 | 9.2 | 11.4 | 3.9 | 1.4 | 1.8 | 2.8 | 7.0 | 7.0 | 7.2 | 13.4 | 13.7 | 9.6 | 10.1 | 0.7 | 0.8 | 0.7 | 1.0 | paratopotype |
| ZUEC 15884 | *P. araguaius* sp. n. | 33.0 | 7.7 | 9.6 | 16.9 | 3.9 | 1.6 | 2.0 | 3.1 | 7.9 | 7.8 | 8.3 | 14.3 | 13.5 | 9.1 | 10.5 | 0.9 | 1.0 | 1.0 | 0.9 | - |
| ZUEC 15885 | *P. araguaius* sp. n. | 32.3 | 6.8 | 9.5 | 15.7 | 4.4 | 1.3 | 1.9 | 2.8 | 6.7 | 7.4 | 8.2 | 13.3 | 12.5 | 8.6 | 10.2 | 1.0 | 1.0 | 0.9 | 1.0 | - |
| ZUEC 15886 | *P. araguaius* sp. n. | 33.1 | 7.1 | 9.7 | 15.9 | 4.1 | 1.8 | 1.9 | 3.0 | 6.6 | 7.8 | 9.0 | 14.5 | 14.0 | 9.7 | 12.0 | 0.9 | 0.9 | 0.9 | 1.0 | - |
| ZUEC 15888 | *P. araguaius* sp. n. | 32.8 | 7.6 | 9.8 | 15.4 | 4.3 | 1.8 | 2.0 | 3.0 | 6.7 | 7.4 | 8.5 | 14.7 | 14.4 | 9.5 | 11.4 | 1.0 | 1.0 | 1.0 | 1.0 | - |
| ZUEC 15887 | *P. araguaius* sp. n. | 32.9 | 7.4 | 9.9 | 15.0 | 4.4 | 1.8 | 1.9 | 2.9 | 7.5 | 7.3 | 8.4 | 14.4 | 14.2 | 9.8 | 10.9 | 1.0 | 1.0 | 0.9 | 1.0 | - |
| ZUEC 21644 | *P. araguaius* sp. n. | 33.6 | 7.8 | 9.7 | 14.9 | 4.1 | 1.6 | 2.1 | 3.0 | 7.2 | 7.5 | 8.1 | 13.5 | 13.6 | 8.7 | 10.7 | 0.8 | 0.9 | 0.9 | 0.9 | - |
| ZUEC 21645 | *P. araguaius* sp. n. | 30.2 | 6.7 | 9.1 | 13.6 | 3.7 | 1.6 | 2.2 | 3.0 | 6.4 | 7.7 | 7.5 | 12.8 | 13.1 | 9.1 | 9.8 | 0.9 | 0.9 | 0.8 | 0.8 | - |
| ZUEC 21647 | *P. araguaius* sp. n. | 31.3 | 7.0 | 9.2 | 13.5 | 3.6 | 1.8 | 2.1 | 3.2 | 7.1 | 7.7 | 7.6 | 14.4 | 13.5 | 9.5 | 9.9 | 0.9 | 1.0 | 0.9 | 1.0 | - |
| CFBH 14321 | *P. araguaius* sp. n. | 32.9 | 7.6 | 10.1 | 17.5 | 4.2 | 1.7 | 2.0 | 3.6 | 8.8 | 8.0 | 8.4 | 14.2 | 14.4 | 9.9 | 11.0 | 0.9 | 1.0 | 1.0 | 1.0 | - |
| CFBH 14322 | *P. araguaius* sp. n. | 33.7 | 7.5 | 10.2 | 16.6 | 4.1 | 1.5 | 1.9 | 3.3 | 8.6 | 7.5 | 8.1 | 14.5 | 14.5 | 9.6 | 11.0 | 1.1 | 1.1 | 1.1 | 1.2 | - |
| CFBH 14367 | *P. araguaius* sp. n. | 29.8 | 6.6 | 9.2 | 14.5 | 4.0 | 1.3 | 1.8 | 3.3 | 6.8 | 7.3 | 7.4 | 12.8 | 12.8 | 8.5 | 10.0 | 0.8 | 0.9 | 0.8 | 1.0 | - |
| CFBH 14368 | *P. araguaius* sp. n. | 30.7 | 7.0 | 9.0 | 14.6 | 4.1 | 1.6 | 1.9 | 2.7 | 6.9 | 7.8 | 7.7 | 12.8 | 13.5 | 9.4 | 10.6 | 0.9 | 0.9 | 0.9 | 0.9 | - |
| CFBH 14369 | *P. araguaius* sp. n. | 28.3 | 6.7 | 8.6 | 14.1 | 3.5 | 1.2 | 1.8 | 2.1 | 6.1 | 6.8 | 6.8 | 12.4 | 11.5 | 8.7 | 9.3 | 0.9 | 1.0 | 0.8 | 0.9 | - |
| CFBH 14370 | *P. araguaius* sp. n. | 29.6 | 6.9 | 9.3 | 14.6 | 3.8 | 1.4 | 2.0 | 2.9 | 7.2 | 7.4 | 7.2 | 12.9 | 13.1 | 8.8 | 10.5 | 1.0 | 1.0 | 1.0 | 0.9 | - |
| CFBH 14406 | *P. araguaius* sp. n. | 33.2 | 8.0 | 9.5 | 16.5 | 3.8 | 2.0 | 2.2 | 3.2 | 7.8 | 7.9 | 8.8 | 15.1 | 15.9 | 9.6 | 10.6 | 0.9 | 0.8 | 0.9 | 1.0 | - |
| ZUEC 13503 | *P. araguaius* sp. n. | 33.9 | 7.3 | 10.3 | 16.2 | 4.0 | 1.8 | 2.2 | 3.6 | 6.4 | 7.8 | 8.9 | 14.5 | 14.2 | 10.0 | 11.6 | 1.1 | 1.1 | 1.0 | 1.0 | - |
| ZUEC 7457 | *P. araguaius* sp. n. | 31.5 | 6.4 | 9.8 | 14.9 | 3.6 | 1.5 | 1.9 | 3.4 | 6.4 | 7.1 | 8.6 | 14.2 | 14.9 | 10.1 | 11.4 | 1.1 | 1.1 | 1.0 | 1.1 | - |
| ZUEC 7458 | *P. araguaius* sp. n. | 32.5 | 7.4 | 10.2 | 15.8 | 3.6 | 1.8 | 2.2 | 3.5 | 7.6 | 7.8 | 8.8 | 14.6 | 15.0 | 9.8 | 11.4 | 1.1 | 1.1 | 1.1 | 1.1 | - |
| AAG-UFU 148 | *P. azureus* | 34.3 | 6.5 | 10.3 | 17.1 | 3.8 | 1.5 | 2.2 | 2.8 | 7.3 | 8.0 | 8.3 | 14.8 | 14.2 | 9.7 | 11.6 | 1.0 | 1.0 | 1.1 | 1.1 | - |
| AAG-UFU 149 | *P. azureus* | 35.2 | 6.5 | 10.3 | 18.4 | 3.8 | 1.5 | 2.3 | 2.7 | 7.8 | 7.8 | 9.2 | 15.0 | 14.2 | 8.9 | 12.1 | 1.0 | 1.0 | 1.0 | 1.0 | - |
| AAG-UFU 150 | *P. azureus* | 34.1 | 6.2 | 10.2 | 16.3 | 3.6 | 1.5 | 1.9 | 3.0 | 7.2 | 7.7 | 9.4 | 15.2 | 15.1 | 9.2 | 11.8 | 1.0 | 1.0 | 0.9 | 1.0 | - |
| AAG-UFU 151 | *P. azureus* | 37.7 | 6.8 | 11.1 | 19.8 | 3.9 | 1.5 | 2.5 | 3.0 | 8.5 | 8.9 | 8.6 | 16.2 | 16.5 | 10.6 | 12.7 | 1.0 | 1.0 | 1.1 | 1.0 | - |
| AAG-UFU 152 | *P. azureus* | 34.4 | 6.5 | 10.3 | 16.7 | 3.8 | 1.5 | 2.5 | 2.8 | 6.8 | 7.1 | 8.4 | 14.7 | 14.0 | 9.1 | 11.6 | 1.0 | 1.1 | 1.2 | 1.2 | - |
| AAG-UFU 153 | *P. azureus* | 34.8 | 6.7 | 10.2 | 17.0 | 3.6 | 1.6 | 2.1 | 2.8 | 7.5 | 7.2 | 9.1 | 14.6 | 14.9 | 9.5 | 11.8 | 0.9 | 1.1 | 1.2 | 1.2 | - |
| MNRJ 61567 | *P. azureus* | 36.6 | 7.1 | 11.0 | 17.7 | 4.0 | 1.6 | 2.9 | 3.8 | 6.6 | 9.8 | 10.6 | 15.6 | 15.3 | 10.8 | 13.1 | 1.0 | 1.1 | 1.0 | 1.1 | - |
| MNRJ 61568 | *P. azureus* | 33.7 | 6.9 | 9.8 | 14.6 | 4.4 | 1.5 | 2.0 | 3.6 | 7.3 | 8.3 | 8.7 | 13.6 | 13.9 | 9.2 | 10.8 | 1.0 | 1.1 | 1.0 | 1.0 | - |
| MNRJ 61569 | *P. azureus* | 33.3 | 6.0 | 9.8 | 15.4 | 4.2 | 1.2 | 2.3 | 3.5 | 7.1 | 8.2 | 9.6 | 14.6 | 14.1 | 9.4 | 11.3 | 1.1 | 1.1 | 1.1 | 1.0 | - |
| MNRJ 61570 | *P. azureus* | 34.4 | 7.2 | 10.7 | 16.5 | 3.7 | 1.9 | 2.4 | 3.8 | 7.4 | 8.0 | 9.4 | 15.0 | 14.0 | 9.9 | 12.2 | 1.0 | 1.1 | 1.1 | 1.1 | - |
| MNRJ 61571 | *P. azureus* | 37.2 | 8.5 | 10.4 | 18.5 | 4.2 | 1.7 | 2.8 | 3.8 | 7.5 | 9.3 | 9.8 | 15.2 | 14.7 | 10.2 | 12.9 | 1.0 | 1.1 | 1.0 | 1.0 | - |
| MNRJ 39995 | *P. azureus* | 39.7 | 7.8 | 10.8 | 18.9 | 4.6 | 1.6 | 2.4 | 3.8 | 6.8 | 8.6 | 10.0 | 14.3 | 15.3 | 9.9 | 12.6 | 1.1 | 1.0 | 1.1 | 1.3 | - |
| MNRJ 13657 | *P. azureus* | 33.1 | 7.9 | 9.8 | 14.4 | 3.7 | 1.7 | 2.1 | 3.6 | 7.1 | 6.5 | 8.8 | 12.8 | 12.8 | 8.9 | 11.4 | 1.0 | 1.1 | 1.4 | 1.2 | topotype |
| MNRJ 13658 | *P. azureus* | 35.0 | 8.3 | 10.8 | 15.7 | 3.8 | 1.8 | 2.1 | 4.0 | 7.3 | 7.6 | 8.6 | 15.2 | 14.5 | 9.5 | 11.2 | 0.7 | 1.0 | 0.9 | 0.8 | topotype |
| MNRJ 13659 | *P. azureus* | 37.4 | 8.0 | 11.2 | 16.1 | 4.1 | 1.7 | 2.5 | 3.8 | 6.4 | 6.8 | 9.1 | 14.3 | 14.0 | 9.7 | 11.6 | 1.1 | 1.2 | 1.1 | 1.1 | topotype |
| MNRJ 13660 | *P. azureus* | 37.7 | 9.8 | 11.3 | 15.2 | 4.2 | 2.0 | 2.4 | 4.3 | 6.1 | 7.4 | 8.9 | 15.2 | 14.8 | 9.0 | 12.0 | 1.2 | 1.1 | 1.2 | 1.1 | topotype |
| MNRJ 13661 | *P. azureus* | 38.2 | 7.8 | 11.4 | 19.3 | 3.9 | 2.3 | 2.3 | 3.8 | 8.0 | 8.7 | 10.1 | 15.5 | 15.9 | 10.4 | 13.0 | 1.2 | 1.1 | 1.2 | 1.2 | topotype |
| MNRJ 13662 | *P. azureus* | 37.6 | 7.8 | 10.6 | 19.3 | 4.2 | 1.6 | 2.6 | 3.6 | 6.4 | 7.5 | 8.8 | 14.2 | 14.6 | 9.7 | 11.8 | 1.1 | 1.1 | 1.0 | 1.0 | topotype |
| MNRJ 13664 | *P. azureus* | 34.7 | 7.7 | 10.3 | 15.2 | 3.8 | 1.7 | 2.1 | 3.8 | 7.2 | 8.7 | 9.2 | 14.6 | 14.3 | 8.9 | 11.1 | 0.7 | 0.7 | 0.8 | 0.7 | topotype |
| MNRJ 13665 | *P. azureus* | 35.3 | 8.1 | 10.4 | 16.0 | 4.4 | 1.9 | 2.2 | 4.0 | 7.4 | 8.1 | 8.7 | 14.2 | 14.4 | 8.9 | 10.8 | 0.9 | 0.7 | 0.8 | 0.7 | topotype |
| MNRJ 13666 | *P. azureus* | 35.5 | 8.4 | 10.4 | 17.1 | 3.5 | 1.6 | 2.4 | 3.3 | 7.0 | 7.6 | 7.8 | 14.0 | 14.0 | 9.6 | 10.9 | 1.0 | 1.1 | 1.0 | 1.1 | topotype |
| MNRJ 13667 | *P. azureus* | 38.3 | 8.8 | 11.2 | 17.3 | 4.4 | 2.3 | 2.3 | 3.9 | 6.6 | 7.1 | 10.1 | 13.7 | 14.6 | 9.4 | 12.0 | 1.0 | 1.0 | 0.7 | 0.8 | topotype |
| MNRJ 13668 | *P. azureus* | 38.9 | 8.0 | 11.3 | 16.6 | 4.3 | 1.7 | 2.5 | 4.3 | 6.8 | 7.2 | 10.0 | 14.8 | 14.6 | 9.3 | 11.5 | 1.2 | 1.0 | 1.5 | 1.2 | topotype |
| MNRJ 13669 | *P. azureus* | 36.7 | 8.2 | 10.7 | 18.3 | 4.5 | 1.5 | 1.9 | 3.8 | 7.1 | 8.2 | 9.5 | 15.6 | 14.4 | 8.3 | 11.7 | 1.2 | 1.0 | 1.1 | 1.1 | topotype |
| MNRJ 13670 | *P. azureus* | 33.7 | 7.8 | 10.7 | 14.1 | 4.1 | 1.7 | 2.8 | 3.3 | 7.0 | 7.4 | 8.2 | 13.7 | 13.5 | 8.8 | 10.3 | 0.8 | 0.7 | 0.7 | 0.9 | topotype |
| ZUEC 18623 | *P. nordestinus* | 34.3 | 6.6 | 10.6 | 14.5 | 4.1 | 1.6 | 2.7 | 3.3 | 7.0 | 7.9 | 9.0 | 16.6 | 15.5 | 9.9 | 11.4 | 0.7 | 0.8 | 0.9 | 0.9 | - |
| ZUEC 18624 | *P. nordestinus* | 33.7 | 7.0 | 9.9 | 14.9 | 3.9 | 1.2 | 2.7 | 3.3 | 6.5 | 8.9 | 8.9 | 14.7 | 15.0 | 9.1 | 11.3 | 0.7 | 0.7 | 0.9 | 0.9 | - |
| ZUEC 18625 | *P. nordestinus* | 32.9 | 7.0 | 10.9 | 15.3 | 4.2 | 1.8 | 2.3 | 3.2 | 7.0 | 7.8 | 8.6 | 15.9 | 14.6 | 9.4 | 10.5 | 1.0 | 0.9 | 0.9 | 1.0 | - |
| ZUEC 18626 | *P. nordestinus* | 34.6 | 6.6 | 10.6 | 17.2 | 4.0 | 1.5 | 2.2 | 3.1 | 7.3 | 7.2 | 8.9 | 14.7 | 13.5 | 9.8 | 11.2 | 0.9 | 0.8 | 0.9 | 1.0 | - |
| ZUEC 19898 | *P. nordestinus* | 37.0 | 6.9 | 11.4 | 16.4 | 4.4 | 1.7 | 2.6 | 3.5 | 6.9 | 9.2 | 9.2 | 15.3 | 14.6 | 9.5 | 11.9 | 1.1 | 1.1 | 1.0 | 1.2 | - |
| ZUEC 19902 | *P. nordestinus* | 36.6 | 6.7 | 10.8 | 17.6 | 3.6 | 1.9 | 2.9 | 3.2 | 6.9 | 8.5 | 10.3 | 15.6 | 15.4 | 10.1 | 12.8 | 1.0 | 1.0 | 1.1 | 1.2 | - |
| ZUEC 19891 | *P. nordestinus* | 36.4 | 7.4 | 11.3 | 17.5 | 4.4 | 1.3 | 2.6 | 3.3 | 7.6 | 9.1 | 9.9 | 15.2 | 14.7 | 9.3 | 12.8 | 1.1 | 1.2 | 1.2 | 1.3 | - |
| ZUEC 19901 | *P. nordestinus* | 33.0 | 7.2 | 10.5 | 14.5 | 4.7 | 1.7 | 2.4 | 3.3 | 7.3 | 9.3 | 9.6 | 15.3 | 15.0 | 9.5 | 11.9 | 1.2 | 1.2 | 1.0 | 1.1 | - |
| ZUEC 19906 | *P. nordestinus* | 34.0 | 6.4 | 10.2 | 15.0 | 4.1 | 1.7 | 2.4 | 3.1 | 7.4 | 8.2 | 8.7 | 13.9 | 14.3 | 9.4 | 11.0 | 0.9 | 1.0 | 1.0 | 0.9 | - |
| ZUEC 19909 | *P. nordestinus* | 36.7 | 6.9 | 11.5 | 16.9 | 4.5 | 1.7 | 2.8 | 3.5 | 8.1 | 8.5 | 11.0 | 16.5 | 16.0 | 10.4 | 13.1 | 0.9 | 1.0 | 0.7 | 0.7 | - |
| ZUEC 19893 | *P. nordestinus* | 35.3 | 6.3 | 11.0 | 16.7 | 4.2 | 1.7 | 2.5 | 3.0 | 7.0 | 8.1 | 8.7 | 15.3 | 14.7 | 9.7 | 11.6 | 0.7 | 0.8 | 0.7 | 0.7 | - |
| ZUEC 19899 | *P. nordestinus* | 37.5 | 7.2 | 10.8 | 19.3 | 4.0 | 1.8 | 2.9 | 3.3 | 6.7 | 7.9 | 9.4 | 15.3 | 15.4 | 10.4 | 11.5 | 0.6 | 0.6 | 0.7 | 0.8 | - |
| ZUEC 19888 | *P. nordestinus* | 37.8 | 6.9 | 11.4 | 19.7 | 4.0 | 1.8 | 2.7 | 3.7 | 7.5 | 7.6 | 9.5 | 16.6 | 15.5 | 11.0 | 12.3 | 0.7 | 0.8 | 0.6 | 0.7 | - |
| ZUEC 19884 | *P. nordestinus* | 37.9 | 7.1 | 11.8 | 18.5 | 4.1 | 2.0 | 2.2 | 3.2 | 7.2 | 8.8 | 11.2 | 16.5 | 15.7 | 10.8 | 12.3 | 0.5 | 0.8 | 1.0 | 1.0 | - |
| ZUEC 19889 | *P. nordestinus* | 36.9 | 6.8 | 10.3 | 17.4 | 3.9 | 1.9 | 2.3 | 2.9 | 7.5 | 8.1 | 8.9 | 14.7 | 14.3 | 9.2 | 11.1 | 0.9 | 1.0 | 0.8 | 0.7 | - |
| ZUEC 19907 | *P. nordestinus* | 34.0 | 7.4 | 10.9 | 14.5 | 4.9 | 2.3 | 2.8 | 3.5 | 7.0 | 8.4 | 9.7 | 14.0 | 14.3 | 10.2 | 11.9 | 1.0 | 0.9 | 1.0 | 1.1 | - |
| ZUEC 19887 | *P. nordestinus* | 39.5 | 8.7 | 12.0 | 19.6 | 4.3 | 2.1 | 3.1 | 3.8 | 7.5 | 9.1 | 10.0 | 16.4 | 15.6 | 11.1 | 12.6 | 1.2 | 1.0 | 0.7 | 0.8 | - |
| ZUEC 19894 | *P. nordestinus* | 36.7 | 8.0 | 11.4 | 18.2 | 4.3 | 1.9 | 2.4 | 3.6 | 6.7 | 8.6 | 9.6 | 15.4 | 14.8 | 9.9 | 12.1 | 0.8 | 0.7 | 0.9 | 0.9 | - |
| ZUEC 19892 | *P. nordestinus* | 35.2 | 7.4 | 11.5 | 18.1 | 4.9 | 2.3 | 2.6 | 3.5 | 7.4 | 9.3 | 9.3 | 15.7 | 15.3 | 10.8 | 11.5 | 0.6 | 0.7 | 0.7 | 0.9 | - |
| ZUEC 19890 | *P. nordestinus* | 38.4 | 8.6 | 12.1 | 19.5 | 4.3 | 2.0 | 3.0 | 3.4 | 8.1 | 8.5 | 9.2 | 16.3 | 15.4 | 10.1 | 12.0 | 0.7 | 0.8 | 0.7 | 0.7 | - |
| ZUEC 19882 | *P. nordestinus* | 38.8 | 8.5 | 12.4 | 19.8 | 4.9 | 2.1 | 2.8 | 3.9 | 8.1 | 8.0 | 10.3 | 15.9 | 15.7 | 10.4 | 12.1 | 0.6 | 0.9 | 0.9 | 1.0 | - |
| ZUEC 19911 | *P. nordestinus* | 35.8 | 8.3 | 11.5 | 18.0 | 4.7 | 1.7 | 2.4 | 3.5 | 7.3 | 8.1 | 9.4 | 15.4 | 14.4 | 9.6 | 11.3 | 1.1 | 1.1 | 1.2 | 1.1 | - |
| ZUEC 19883 | *P. nordestinus* | 35.7 | 7.5 | 11.5 | 18.7 | 3.8 | 2.1 | 2.9 | 3.5 | 7.3 | 8.0 | 10.3 | 15.0 | 14.7 | 9.8 | 10.5 | 0.8 | 1.0 | 1.0 | 1.0 | - |
| ZUEC 19885 | *P. nordestinus* | 36.5 | 8.0 | 11.7 | 16.7 | 4.4 | 1.7 | 2.4 | 3.2 | 7.3 | 7.5 | 9.2 | 15.0 | 15.7 | 9.8 | 11.4 | 0.7 | 0.8 | 0.8 | 0.8 | - |
| ZUEC 19900 | *P. nordestinus* | 37.1 | 7.7 | 11.4 | 17.0 | 4.2 | 2.1 | 2.4 | 3.9 | 7.4 | 9.2 | 9.9 | 15.6 | 14.9 | 9.5 | 12.9 | 0.9 | 0.6 | 0.9 | 1.1 | - |
| ZUEC 19895 | *P. nordestinus* | 34.4 | 7.3 | 10.6 | 17.2 | 3.4 | 1.4 | 2.5 | 3.2 | 7.7 | 7.9 | 9.5 | 14.9 | 14.7 | 9.1 | 11.3 | 0.8 | 0.9 | 1.0 | 1.0 | - |
| ZUEC 19903 | *P. nordestinus* | 36.5 | 8.0 | 11.0 | 16.5 | 3.6 | 1.7 | 2.5 | 3.0 | 7.1 | 7.9 | 9.0 | 14.2 | 14.3 | 9.5 | 11.7 | 1.1 | 1.3 | 1.0 | 1.0 | - |
| ZUEC 19913 | *P. nordestinus* | 37.6 | 7.4 | 11.9 | 17.5 | 4.9 | 2.0 | 2.3 | 3.7 | 7.3 | 6.9 | 9.6 | 14.6 | 15.7 | 11.1 | 12.0 | 1.1 | 1.2 | 1.1 | 1.1 | - |
| ZUEC 19897 | *P. nordestinus* | 36.2 | 7.9 | 10.9 | 17.2 | 3.6 | 1.8 | 2.4 | 3.4 | 7.3 | 8.9 | 10.3 | 15.6 | 14.7 | 9.6 | 11.6 | 1.2 | 1.2 | 1.2 | 1.4 | - |
| ZUEC 19912 | *P. nordestinus* | 36.0 | 7.6 | 11.8 | 17.3 | 4.0 | 2.2 | 2.6 | 3.7 | 7.3 | 8.8 | 10.1 | 15.8 | 15.3 | 10.6 | 11.2 | 1.1 | 1.2 | 1.3 | 1.3 | - |
| ZUEC 19908 | *P. nordestinus* | 37.7 | 7.4 | 11.2 | 19.9 | 4.3 | 2.0 | 2.2 | 3.1 | 7.8 | 8.8 | 9.3 | 15.4 | 14.2 | 9.7 | 12.1 | 0.8 | 0.7 | 0.8 | 0.8 | - |
| MNRJ 13607 | *P. nordestinus* | 34.3 | 7.2 | 10.7 | 17.2 | 4.4 | 1.5 | 2.4 | 3.5 | 7.0 | 7.6 | 9.2 | 15.0 | 14.7 | 9.8 | 11.3 | 1.0 | 1.1 | 1.0 | 1.0 | holotype |
| MNRJ 13602 | *P. nordestinus* | 37.1 | 7.7 | 11.1 | 18.7 | 4.3 | 1.5 | 2.9 | 3.5 | 8.1 | 8.3 | 10.0 | 14.9 | 15.0 | 10.5 | 12.0 | 1.0 | 1.0 | 1.1 | 1.1 | paratopotype |
| MNRJ 13603 | *P. nordestinus* | 35.8 | 7.7 | 10.9 | 17.7 | 4.1 | 1.4 | 2.5 | 3.5 | 7.9 | 8.0 | 8.4 | 15.3 | 15.1 | 9.6 | 10.4 | 1.0 | 0.9 | 0.9 | 1.0 | paratopotype |
| MNRJ 13604 | *P. nordestinus* | 35.3 | 7.5 | 10.8 | 14.5 | 4.3 | 1.4 | 2.4 | 3.6 | 6.9 | 7.5 | 9.1 | 14.7 | 13.9 | 9.1 | 11.2 | 1.0 | 1.1 | 1.1 | 1.2 | paratopotype |
| MNRJ 13605 | *P. nordestinus* | 38.1 | 7.7 | 12.7 | 18.9 | 4.1 | 2.0 | 2.5 | 3.8 | 7.8 | 9.2 | 8.9 | 15.6 | 15.7 | 10.7 | 12.1 | 1.0 | 1.0 | 1.1 | 1.2 | paratopotype |
| MNRJ 13606 | *P. nordestinus* | 35.2 | 7.2 | 10.8 | 15.9 | 3.9 | 1.8 | 2.5 | 3.5 | 7.4 | 7.5 | 9.3 | 15.2 | 14.7 | 9.9 | 11.3 | 1.1 | 1.1 | 1.1 | 1.0 | paratopotype |
| MNRJ 13608 | *P. nordestinus* | 35.1 | 7.2 | 10.8 | 17.5 | 3.9 | 1.5 | 2.4 | 3.4 | 7.4 | 7.6 | 9.4 | 13.7 | 13.6 | 9.1 | 11.5 | 1.1 | 1.0 | 1.0 | 1.1 | paratopotype |
| MNRJ 13609 | *P. nordestinus* | 36.8 | 8.2 | 10.8 | 17.3 | 3.9 | 1.6 | 2.6 | 3.5 | 7.2 | 8.3 | 9.0 | 13.9 | 14.0 | 9.4 | 11.4 | 1.0 | 0.9 | 1.1 | 1.2 | paratopotype |
| MNRJ 13610 | *P. nordestinus* | 35.9 | 7.6 | 11.1 | 16.5 | 4.2 | 1.8 | 2.2 | 3.4 | 7.1 | 7.8 | 9.4 | 15.2 | 14.4 | 9.1 | 11.7 | 1.1 | 1.0 | 1.1 | 1.1 | paratopotype |
| MNRJ 13611 | *P. nordestinus* | 37.5 | 7.8 | 11.5 | 17.2 | 4.4 | 1.7 | 2.6 | 3.3 | 7.5 | 8.7 | 8.5 | 15.8 | 14.7 | 10.1 | 11.9 | 1.0 | 1.0 | 1.0 | 1.1 | paratopotype |
| MNRJ 60097 | *P. nordestinus* | 34.0 | 7.8 | 10.3 | 15.8 | 3.3 | 2.0 | 2.4 | 3.4 | 7.0 | 8.1 | 8.7 | 14.6 | 13.8 | 9.9 | 11.0 | 1.1 | 1.0 | 1.2 | 1.2 | paratopotype |
| MNRJ 13598 | *P. nordestinus* | 34.4 | 6.9 | 10.2 | 16.1 | 3.4 | 1.6 | 2.6 | 3.3 | 6.6 | 7.6 | 8.6 | 13.6 | 13.4 | 9.3 | 11.2 | 1.0 | 1.1 | 1.1 | 1.2 | paratopotype |
| MNRJ 13599 | *P. nordestinus* | 42.5 | 9.0 | 11.4 | 18.3 | 4.9 | 1.7 | 2.5 | 4.0 | 6.7 | 7.9 | 9.2 | 15.1 | 14.9 | 10.7 | 11.8 | 1.1 | 1.1 | 1.1 | 1.4 | paratopotype |
| MNRJ 13600 | *P. nordestinus* | 35.2 | 7.0 | 10.6 | 14.9 | 3.7 | 2.3 | 2.4 | 3.4 | 6.5 | 7.5 | 8.7 | 14.1 | 13.8 | 9.3 | 11.1 | 1.0 | 1.1 | 1.1 | 1.4 | paratopotype |
| MNRJ 13601 | *P. nordestinus* | 40.9 | 8.1 | 11.8 | 20.8 | 4.4 | 2.1 | 2.8 | 3.9 | 7.3 | 9.3 | 10.3 | 16.4 | 16.2 | 10.7 | 12.7 | 1.4 | 1.2 | 1.4 | 1.5 | paratopotype |
| MNRJ 35223 | *P. nordestinus* | 36.4 | 9.7 | 11.1 | 17.9 | 4.5 | 1.7 | 2.8 | 3.8 | 8.0 | 8.1 | 9.3 | 15.3 | 15.3 | 9.5 | 12.0 | 1.1 | 1.0 | 1.1 | 1.0 | paratopotype |
| MNRJ 35224 | *P. nordestinus* | 36.2 | 8.2 | 11.1 | 17.5 | 4.5 | 1.7 | 2.7 | 3.1 | 7.7 | 8.4 | 9.4 | 15.0 | 15.3 | 9.8 | 12.0 | 1.1 | 1.1 | 1.1 | 1.2 | paratopotype |
| MNRJ 35225 | *P. nordestinus* | 37.3 | 7.6 | 10.9 | 19.1 | 4.2 | 1.3 | 2.6 | 3.7 | 8.5 | 8.2 | 9.5 | 15.3 | 15.7 | 9.8 | 11.6 | 1.0 | 1.2 | 1.2 | 1.2 | paratopotype |
| MNRJ 35226 | *P. nordestinus* | 34.3 | 7.5 | 10.4 | 17.1 | 3.8 | 1.4 | 2.6 | 3.7 | 7.6 | 8.2 | 8.7 | 14.5 | 14.3 | 8.9 | 10.9 | 1.0 | 1.1 | 1.2 | 1.2 | paratopotype |
| MNRJ 35227 | *P. nordestinus* | 36.1 | 7.4 | 10.6 | 17.4 | 3.5 | 1.7 | 2.4 | 3.5 | 7.0 | 8.3 | 8.5 | 13.7 | 13.8 | 8.8 | 11.0 | 1.2 | 1.0 | 1.1 | 1.1 | paratopotype |
| MNRJ 35228 | *P. nordestinus* | 36.0 | 7.6 | 10.3 | 16.3 | 4.1 | 1.7 | 2.0 | 3.1 | 6.6 | 7.9 | 8.0 | 13.6 | 14.3 | 9.7 | 11.3 | 1.0 | 1.0 | 1.0 | 1.1 | paratopotype |

**Abbreviation to collections:** ZUEC (Museu de Zoologia da Unicamp, Universidade Estadual de Campinas, Brazil); AAG-UFU (Collection of frogs of the Museu de Biodiversidade do Cerrado, Universidade Federal de Uberlândia, Brazil); MNRJ (Museu Nacional do Rio de Janeiro, Universidade Federal do Rio de Janeiro, Brazil); CFBH (Célio F. B. Haddad, Universidade Estadual Paulista, Brazil). Morphometric traits: snout-vent length (SVL), hand length (HAL), forearm length (FAL), thigh length (THL), foot length (FL), head length (HL), head width (HW), eye diameter (ED), internarial distance (IND), tibia length (TL) (=shank length), tympanum diameter (TD), and eye-nostril distance (END), upper arm length (UAL), tarsus length (TAL), the disc diameters of third finger (3FD), fourth finger (4FD), fourth toe (4TD), fifth toe (5TD), and axilla-groin length (AGL).
